# Oviducal gland transcriptomics of *Octopus maya* through physiological stages and the negative effects of temperature on fertilization

**DOI:** 10.7717/peerj.12895

**Published:** 2022-03-30

**Authors:** Oscar E. Juárez, Lousiana Arreola-Meraz, Edna Sánchez-Castrejón, Omar Hernando Avila-Poveda, Laura L. López-Galindo, Carlos Rosas, Clara E. Galindo-Sánchez

**Affiliations:** 1 Departamento de Biotecnología Marina, Centro de Investigación Científica y de Educación Superior de Ensenada, Baja California, Ensenada, Baja California, México; 2 Facultad de Ciencias del Mar, Universidad Autónoma de Sinaloa, Mazatlán, Sinaloa, México; 3 Programa Investigadoras e Investigadores por México, Consejo Nacional de Ciencia y Tecnología, Ciudad de México, México; 4 Instituto de Investigaciones Oceanológicas, Universidad Autónoma de Baja California, Ensenada, Baja California, México; 5 Unidad Multidisciplinaria de Docencia e Investigación - Sisal, Facultad de Ciencias, Universidad Nacional Autónoma de México, Sisal, Yucatán, México

**Keywords:** Egg-laying, Fertilization, Gamete recognition proteins, RNA-Seq, Thermal stress

## Abstract

**Background:**

Elevated temperatures reduce fertilization and egg-laying rates in the octopus species. However, the molecular mechanisms that control the onset of fertilization and egg-laying in the octopus’ oviducal gland are still unclear; and the effect of temperature on the expression of key reproductive genes is unknown. This study aims to better understand the molecular bases of octopus fertilization and egg-laying, and how they are affected by elevated temperatures.

**Method:**

RNA-seq of oviducal glands was performed for samples before, during, and after fertilization and their transcriptomic profiles were compared. Also, at the fertilization stage, the optimal and thermal-stress conditions were contrasted. Expression levels of key reproductive genes were validated *via* RT-qPCR.

**Results:**

In mated females before egg-laying, genes required for the synthesis of spermine, spermidine, which may prevent premature fertilization, and the myomodulin neuropeptide were upregulated. Among the genes with higher expression at the fertilization stage, we found those encoding the receptors of serotonin, dopamine, and progesterone; genes involved in the assembly and motility of the sperm flagellum; genes that participate in the interaction between male and female gametes; and genes associated with the synthesis of eggshell mucoproteins. At temperatures above the optimal range for reproduction, mated females reduced the fertilization rate. This response coincided with the upregulation of myomodulin and APGW-amide neuropeptides. Also, genes associated with fertilization like LGALS3, VWC2, and Pcsk1 were downregulated at elevated temperatures. Similarly, in senescent females, genes involved in fertilization were downregulated but those involved in the metabolism of steroid hormones like SRD5A1 were highly expressed.

## Introduction

The endemic *Octopus maya* from the Yucatan Continental Shelf is becoming a good model to study the eco-physiological adaptations to environmental challenges, such as ocean warming (Juárez et al., 2015, 2016; Sanchez-García et al., 2017; López-Galindo et al., 2019a; Meza-Buendía et al., 2021). This ectothermic species is adapted to a narrow temperature range: reproductive events and embryonic development have an upper thermal limit around 27 °C; below that temperature—and optimally around 24 °C—these processes take place successfully. Therefore, reproductive events occur typically on winter and during upwelling pulses when sea temperatures are cooler on this region (Juárez et al., 2015; Caamal-Monsreal et al., 2016; Avila-Poveda et al., 2016; Angeles-Gonzalez et al., 2017; Sanchez-García et al., 2017; Pascual et al., 2019; López-Galindo et al., 2019a, 2019b). However, higher temperatures have been reported in its distribution area in recent years, mainly in summer, which may negatively affect the physiological condition and reproductive success in *O. maya* males and females (Juárez et al., 2015, 2016; Angeles-Gonzalez et al., 2017; Pascual et al., 2019; López-Galindo et al., 2019a, 2019b). For instance, gene expression patterns in the testis of thermally stressed *O. maya* males were associated with physiological deficiencies and low motility of the spermatozoa (López-Galindo et al., 2019b). In *O. maya* females, the exposure to temperatures above 27 °C significantly reduced the ova production, yolk amount, fertilization, and egg-laying rates (Juárez et al., 2015). Moreover, the effect of thermal stress on mated females substantially reduced the hatchling survival, and hatchling growth rate. Therefore, the ability of *O. maya* females to store spermatozoa and delay fertilization until thermal conditions become favorable may be an adaptation that prevents low survival of the offspring at high temperatures (Juárez et al., 2015, 2016). However, the molecular mechanisms controlling the onset of fertilization and egg-laying are still unclear.

*Octopus maya* is a semelparous species with only one reproductive event (egg-laying) in its lifetime and near the end of life when the female dies after the eggs hatch (Van Heukelem, 1983). In *Octopus*, the reproductive system consists of a gonad oval with two tubular oviducts and one oviducal gland (OvG) arranged halfway along each oviduct (Wells & Wells, 1977; Arkhipkin, 1992). In general terms, the OvG is involved in spermatozoa storage in spermathecae (Mangold, 1987; Marian, 2011, 2015), ova fertilization, and two critical activities: the production of cement by the peripheral gland and the cement polymerization in the central gland (Mangold, von Boletzky & Frösch, 1971; Froesch & Marthy, 1975; Wells & Wells, 1977). This cement is used to stick the eggs together in strings and attach them to the walls or roof of the female’s shelter (Froesch & Marthy, 1975; Wells, 1978).

During mating, the passage of spermatozoa into the OvG towards the spermathecae does not imply fertilization; spermatozoa are stored in the spermathecae and used later in batches (Mangold, 1987; Marian, 2015; Sato, 2021). In wild and laboratory conditions, females continue mating and storing spermatozoa as the OvG enlarges (Froesch & Marthy, 1975; López-Galindo et al., 2019a). Fertilization is internal: mature ova descend one by one from their follicular sheath to the proximal oviduct; simultaneously, spermatozoa are released from the spermathecae into the oviduct of the OvG for fertilization to occur (Mangold, 1987). Then, the egg-laying female stops hunting and feeding, to dedicate herself exclusively to the care and protection of her egg mass; therefore, the onset of egg-laying depends on the energetic budget of the female, because she needs enough energy reserves to perform the oviposition and egg incubation—which takes around 45 days—without food (O’Dor & Wells, 1978; Di Cristo, 2013; Juárez et al., 2015; Meza-Buendía et al., 2021). Egg-incubation coincides with the onset of senescence, where a general physiological decline and diseases occur, as part of a natural process that precedes the octopus death; females die just after the newborns’ hatch (Anderson, Wood & Byrne, 2002). Physical deterioration of distinct organs occurs in the senescent female’s body, including the depletion of reproductive organs, where the OvG drastically shrinks (Olivares et al., 2017).

The OvG plays a crucial role in the reproductive strategy, thus deserves more consideration in the study of octopus reproductive success (Olivares et al., 2017; Sato, 2021). In the present study, the transcriptomic profiles of OvG at different physiological stages were analyzed to understand the molecular mechanisms controlling the onset of fertilization and egg-laying in *O. may*a. We also evaluated the effect of temperature on the expression of key reproductive genes at the fertilization stage.

## Materials and Methods

### Ethics statement

In this study, octopuses were anesthetized with ethanol 3% in seawater at experimental temperatures (Estefanell et al., 2011; Gleadall, 2013) to enable humane killing (Andrews et al., 2013) in consideration of ethical protocols (Mather & Anderson, 2007), and the animals’ welfare during manipulations (Moltschaniwskyj et al., 2007; Winlow et al., 2018), all previous takes into account the nociception in aquatic invertebrates (Sneddon, 2015). Our protocols were approved by the experimental Animal Ethics Committee of the Faculty of Chemistry at Universidad Nacional Autónoma de México (Permit number: Oficio/FQ/CICUAL/099/15) and following the ethical recommendations for the humanitarian killing of animals as established under Mexican law (NOM-033-SAG/ZOO-2014 derogating to NOM-033-ZOO-1995) and following as closely as possible the five R’s Principle in invertebrates (Crespi-Abril & Rubilar, 2021).

### Capture and acclimation of octopuses

**Figure 1:**
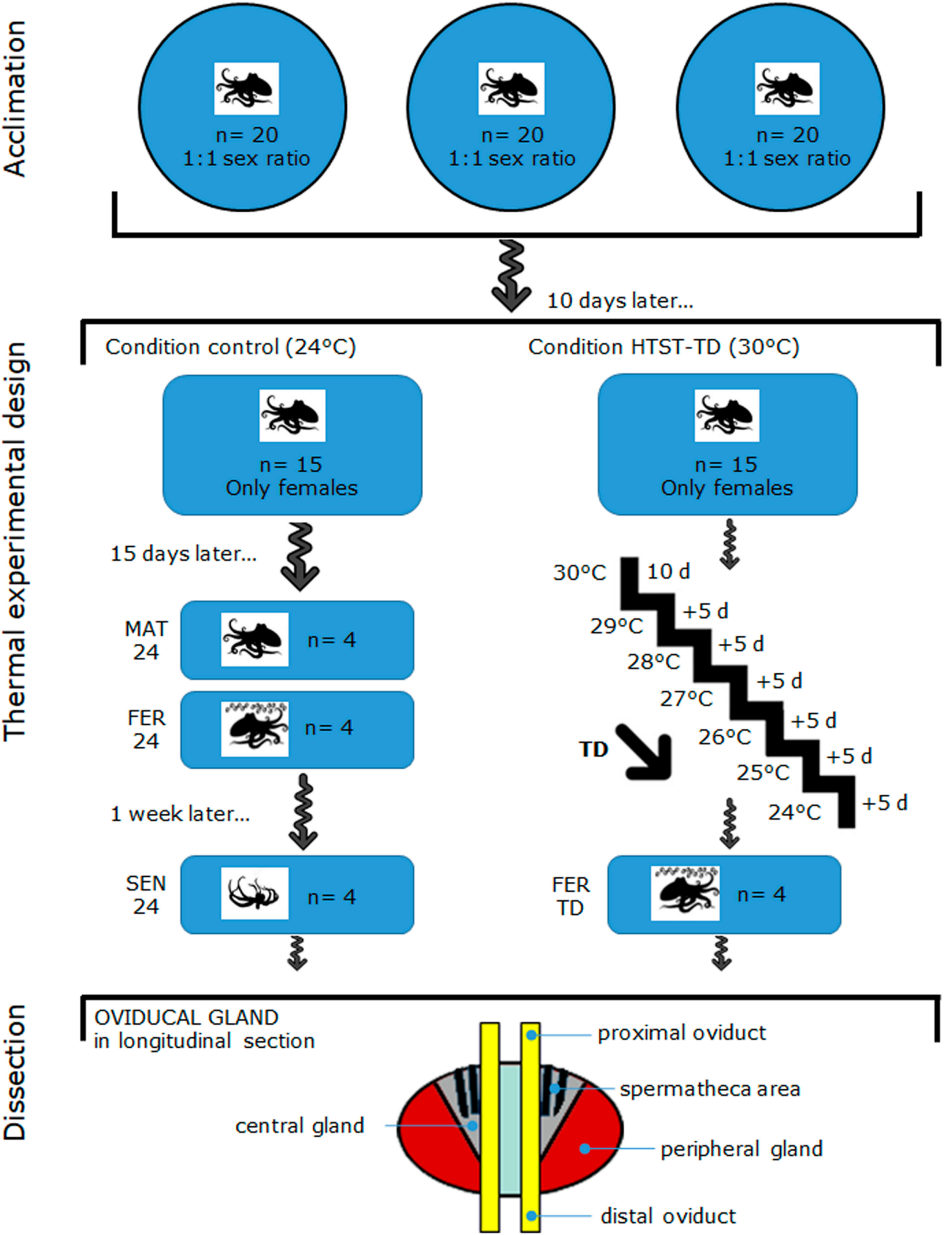
Schematic drawing of the acclimation, thermal experimental design, and oviducal gland dissection in *O. maya*. MAT24 for mated females at 24 °C, FER24 for fertilization in females at 24 °C, SEN24 for senescent females at 24 °C and, FER-TD for fertilization in females from the high-temperature shock treatment with temperature decrease (HTST-TD).

Female (*n* = 30) and male (*n* = 30) octopuses with a body mass that ranged between 400–600 g were captured off the coast of Sisal Yucatán, México, by an artisanal fishing fleet. This *O. maya* size range assumes that they are reproductively mature, and even some females have already collected sperm in their OvG during the process of maturation (Avila-Poveda et al., 2016; Angeles-Gonzalez et al., 2017; Markaida, Méndez-Loeza & Rosales-Raya, 2017). Captured octopuses were transported to the Experimental Cephalopod Production Unit at the Unidad Multidisciplinaria de Docencia e Investigación (UMDI-UNAM), Sisal, Yucatan, Mexico. Figure 1 (on top) shows how the octopuses were acclimated for 10 d in three outdoor ponds (6 m diameter) containing aerated natural seawater (oxygen level maintained to 5.5 ± 0.5 mg L^−1^) at 25 ± 1 °C and a rate of 20 individuals per pond with a sex ratio of 1:1 (Rosas et al., 2014) following the acclimation protocol of López-Galindo et al. (2019a). Octopuses were observed daily at 6:00 AM, 12:00 PM, and 6:00 PM for 1 h. Mating was observed since the first day of acclimation which was confirmed by observing a male inserting his hectocotylus (mating arm) into the female mantle cavity to deposit spermatophores. During the 10-d acclimation period, all the females mated at least once.

### Experimental design and thermal conditions

Figure 1 illustrates the thermal experimental design and oviducal gland dissection after the acclimation, which are subsequently described in detail: on day 11, mated females were distributed randomly (coin toss) into two recirculating aquaculture systems, one for the Control condition (*n* = 15) and another for the thermal challenge (*n* = 15). Females were individually reared in 80 L tanks for 40 d in both Control and Treatment conditions (from day 11 to 51). Each tank contained a fiberglass box that served as a refuge and for egg-laying. Physio-chemical parameters (dissolved oxygen, temperature, salinity, and pH) of individual tanks were monitored twice daily to ensure homogeneous conditions within each treatment and minimize potential confounders. In the Control condition, females were reared at 24 °C, which is the best condition for egg-laying (Rosas et al., 2014; Juárez et al., 2015). The thermal challenge consisted of a high-temperature shock treatment with a temperature decrease (HTST-TD), which was performed following the protocol of Juárez et al. (2015), where mated females were initially exposed to a stress temperature of 30 °C for 10 d (from day 11 to day 21), then they were exposed 5 d at temperatures from 29 to 24 (decreasing of 1 °C every 5 days) (Fig. 1). After the heat shock of 30 °C, the system was cooled down to induce the egg-laying (Juárez et al., 2015).

### Egg-laying and fertilization rates

Through the experiment, the average number of eggs per spawn was calculated and the fertilization was visually inspected in each treatment following the protocol described in Juárez et al. (2015) including all the females that laid eggs in each experimental condition. The egg-laying rates were normalized by dividing the number of eggs per spawn by the females’ weight in grams. Fertilization rates were expressed as the proportion of eggs containing embryos at the end of the incubation process, from the total eggs laid per female. Statistical differences in egg-laying rates and fertilization rates between the Control and the HTST-TD were evaluated by implementing Student t-tests with a statistical significance of *P* < 0.05.

### Sampling and dissection of oviducal glands

We replicated the sampling schedule implemented by Juárez et al. (2015): sampling began after 2 weeks of exposure to the experimental conditions, on day 26. Females that started the egg-laying before that day were not considered for further analysis. In the Control condition, four females in the mated stage (MAT24, *n* = 4) were sampled on day 26. The mated stage was characterized by an OvG that received spermatophores and stored spermatozoa in the spermathecae, according to the previous observation of the male(s) inserting the hectocotylus arm into the female’s mantle cavity. The onset of egg-laying—which coincides with fertilization and precedes the senescence—was unpredictable for each female. Therefore, a female was considered at the fertilization stage as soon as the egg-laying was detected during the daily monitoring. At that moment, females were sheltered in their nests and stopped feeding, whereas the OvG was presumably releasing sperm from the spermatheca and producing secretions from its glands. The next egg-laying female detected was sampled 1 week after she started laying her eggs, which represented the senescence stage. This stage was characterized by an OvG that entered in physiological (without evident secretions) and anatomical deterioration due to a phase of programmed cell death; besides, females were also near the end of their life. This was repeated until obtaining four samples for each stage (FER24, *n* = 4; SEN24, *n* = 4).

In the HTST-TD, females observed in the fertilization stage (FER-TD, *n* = 4) were sampled from day 26 to day 51 (Fig. 1). Before OvG dissection, animals were anesthetized by keeping them in ethanol–seawater (3%) solution for up to 4 min as indicated in the Ethics statement. Subsequently, the reproductive system was dissected from each octopus for each experimental condition (MAT24, FER24, SEN24, and FER-TD) and OvG samples were cut from the peripheral gland, central gland, and spermatheca area, which were mixed trying to obtain representative samples of the whole oviducal gland (Fig. 1 at the bottom), and finally preserved in RNAlater solution (Thermo Fisher) at 4 °C. Once all the samples were obtained, they were sent to the Laborario de Genómica Funcional de Organismos Marinos of CICESE in Ensenada, BC, Mexico where they were stored at −70 °C until RNA extraction.

### RNA sequencing

RNA extraction, quantitation, and quality check were performed using the protocol, reagents, and instruments described in López-Galindo et al. (2019b) and Juárez et al. (2019), starting with 20–30 mg of every whole gland sample. For each experimental condition (MAT24, FER24, SEN24, and FER-TD), a pooled sample was prepared which consisted of 100 ng of RNA from four different individuals (*n* = 4). Paired-end libraries of complementary DNA (cDNA) were prepared for each pool using the TruSeq DNA Sample Preparation Kit v2 (Illumina, San Diego, CA, USA), following the manufacturer’s protocol. Libraries were sequenced in the MiSeq system (Illumina, San Diego, CA, USA) to obtain reads of 150 bp long. Libraries and sequencing were conducted without the knowledge of treatment allocation of the pooled samples (blinding).

### Bioinformatic workflow

The quality reports of raw sequence data were obtained with FastQC v0.11.6 (Babraham Bioinformatics, Babraham, UK). Low-quality reads, ambiguous nucleotides, and sequencing adaptors were removed using Trimmomatic v0.35 software (Bolger, Lohse & Usadel, 2014). The transcriptome was *de novo* assembled using Trinity v2.4.0 (Grabherr et al., 2011) and was deposited at DDBJ/EMBL/GenBank database under the accession GJEO00000000. TransDecoder (Haas et al., 2013) was implemented to predict the open reading frames (ORFs) and coding sequences (CDS) of each transcript (with a minimum length of 50 amino acids), which were annotated using BLASTx searches (Camacho et al., 2009) in the UniProt database. Differential gene expression was analyzed implementing pairwise comparisons among the physiological stages in the Control condition (*i.e*., MAT24 to FER24, MAT24 to SEN24, FER24 to SEN24), then the representative DEGs for each stage included those obtained against the other two stages; for example, representative DEGs of MAT24 included those obtained against FER24 plus those against SEN24, removing the redundancy. The fertilization stages of the Control and the HTST-TD were compared (FER24 against FER-TD); for this, Bowtie2 v2.3.2 (Langmead & Salzberg, 2012) was used to align the reads of each library on the assembled transcriptome; RSEM v1.3.0 (Li & Dewey, 2011) to quantify transcript abundance of each library; and edgeR (Bioconductor) for identification of differential expression at isoform level (Robinson, McCarthy & Smyth, 2009). UniProt IDs of differentially expressed genes (DEGs) were used for enrichment analysis of gene ontology (GO) terms and metabolic pathways from the Kyoto Encyclopedia of Genes and Genomes (KEGG) (Kanehisa et al., 2012) with DAVID v6.8 (Huang, Sherman & Lempicki, 2009). DEGs included in the best-represented GO and KEGG categories and those associated with the regulation of OvG activity (Di Cosmo, Di Cristo & Paolucci, 2001; Iwakoshi-Ukena et al., 2004; Di Cristo & Di Cosmo, 2007; Minakata et al., 2009) were selected for cluster analysis and plotted in heatmaps using R software (R Development Core Team, 2010). The analyses were performed following the scripts and parameters set and statistical significance implemented by Juárez et al. (2019) and López-Galindo et al. (2019b).

### Validation of gene expression *via* RT-qPCR

To validate expression levels found by bioinformatic methods, DEGs with relevant reproductive functions were selected to estimate their expression by using RT-qPCR. Five potential reference genes were evaluated: V-type proton ATPase subunit d (VATD), Elongation factor 1-beta (EF1β), Gelsolin-like protein 2, Heterogeneous nuclear ribonucleoprotein D (HNR), and Ribosomal Protein L6 (RPL6). Specific primers for the target and reference genes were designed using Primer3 (Untergasser et al., 2012). For each stage, additional replicate samples were obtained from animals under the same experimental conditions MAT24 (*n* = 4), FER24 (*n* = 3), SEN24 (*n* = 3), FER-TD (*n* = 3), including MAT-TD (*n* = 5) and SEN-TD (*n* = 3). RNAs were extracted as mentioned previously and treated with the RQ1 RNase-free DNase (Promega, Madison, WI, USA) according to the manufacturer’s protocol. The cDNA was synthesized using the Improm II Reverse Transcription System (Promega, Madison, WI, USA) following the manufacturer’s instructions starting with 2 µg of RNA of each sample. The primer amplification efficiency was estimated following the procedure of Bustin et al. (2009). The RT-qPCR reactions were performed by triplicate following the protocol of López-Galindo et al. (2019b) without knowledge of group allocation of the samples (blind analysis). The stability of reference genes was evaluated using RefFinder (Xie et al., 2012). The relative expression of target genes was estimated following the method proposed by Hellemans et al. (2007). To assess if gene expression levels were significantly affected by the OvG physiological stages and the HTST-TD condition a two-way ANOVA was performed followed by Tukey’s HSD test. A Spearman correlation for RNA-seq and RT-qPCR expression values was performed. Analyses were performed using STATISTICA 8.0 (StatSoft, Tulsa, OK, USA), and a statistically significant difference was accepted at *P* < 0.05.

## Results

In the Control condition, all females laid eggs at a temperature of 24 ± 1 °C, while in the HTST-TD, females did not lay eggs at temperatures around 30 °C, they did it until the rearing system reached temperatures below 28 °C at an average temperature of 26.1 °C. Significant differences in the egg-laying rates (*P* = 8.12E−5) and fertilization rates (*P* = 0.0012) were obtained between the Control and the HTST-TD. In the Control condition, the average number of eggs laid was three times higher and the average number of eggs laid per female’s mass (grams) was two times higher than those observed in the HTST-TD. The average fertilization rate in the HTST-TD was 40.6 with an SD of 26.7, while in the Control condition the average fertilization rate was 72.5 with an SD of 4.0.

### Sequencing and transcriptome assembly

The RNA sequencing generated a total of 21,664,484 paired reads, with an average of 5,416,121 paired reads per library. The raw sequence reads were deposited in the NCBI-SRA database (accession numbers: SRR13512014–SRR13512017). After the quality filter, 19,969,819 paired reads survived (92.17%), which were utilized for transcriptome assembly. The transcriptome consisted of 61,575 contigs with N50 of 593 and 32,348,437 assembled bases. A total of 36,136 coding sequences (CDS) were detected (Table 1).

**Table 1:** Sequencing and reads assembly of cDNA libraries from oviducal glands of *Octopus maya* at different conditions.

Libraries	Number of paired reads
MAT24	5,872,593
FER24	4,931,726
SEN24	5,507,769
FER-TD	5,352,396
Average	5,416,121
Total	21,664,484
Passed QC filter	19,969,819
Transcriptome assembly	quantity
Contigs	61,575
Coding sequences	36,136
Contig length N50, nucleotides	593
Total assembled nucleotides	32,348,437

**Note:**

MAT, mated; FER, fertilization; SEN, senescence; number 24 indicates 24 °C; TD, heat shock treatment with temperature decrease.

### Differential expression analysis

**Figure 2:**
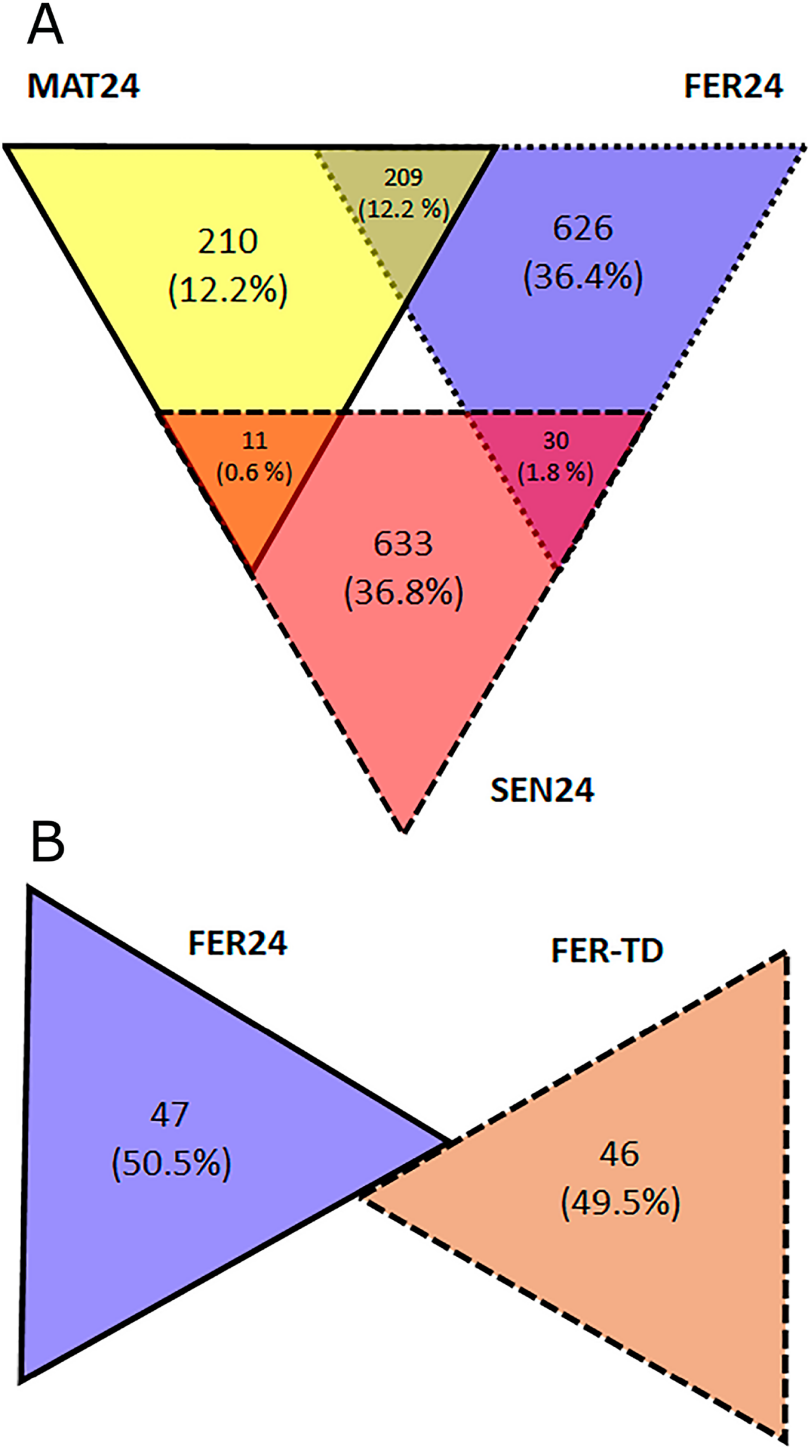
Diagrams representing the number of upregulated transcripts in pairwise comparisons among (A) physiological stages; and (B) between thermal conditions of *O. maya* oviducal glands.

In the Control condition, a total of 1,719 transcripts were differentially expressed including all the pairwise comparisons among the physiological stages, of which, 210 showed the highest expression in MAT24, 626 in FER24, and 633 in SEN24. Gene upregulation was shared between stage pairs: 209 transcripts by the MAT24-FER24 pair, 11 transcripts by MAT24 and SEN24, and 30 transcripts by the FER24-SEN24 pair. In the comparison between FER24 and FER-TD, 93 transcripts showed differential expression, of which 47 showed higher expression in FER24 and 46 in FER-TD (Fig. 2).

### Functional annotation and enrichment analysis

A total of 15,145 transcripts showed significant BLASTx-hit with the UniProt database, of which 12,678 obtained GO annotations. These annotations were analyzed to find enriched biological processes in the transcriptome (Table 2). The higher enrichment was obtained for cilium-dependent cell motility and axoneme assembly, including genes like SPEF2, TTLL1, TTLL3, DRC1, CCDC39, RSPH4A, DNAH1, SPAG16, and LRGUK.

**Table 2:** Functional enrichment of biological processes for the whole oviducal gland transcriptome of *Octopus maya*.

GO	Term	*P*-value	FE
GO:0060285	cilium-dependent cell motility	1.39E−04	5.51
GO:0035082	axoneme assembly	7.24E−04	4.01
GO:0000722	telomere maintenance *via* recombination	4.69E−04	3.08
GO:0006383	transcription from RNA polymerase III promoter	6.50E−04	2.99
GO:0075733	intracellular transport of virus	5.96E−04	2.53
GO:0006396	RNA processing	4.18E−06	2.42
GO:0090630	activation of GTPase activity	1.50E−04	2.30
GO:0006283	transcription-coupled nucleotide-excision repair	4.20E−04	2.25
GO:0016925	protein sumoylation	2.09E−04	2.01
GO:0060271	cilium morphogenesis	5.97E−05	2.01

**Note:**

GO, gene ontology; FE, fold enrichment.

In mated females of the Control condition (MAT24), DEGs enriched biological processes like the fat-soluble vitamin metabolic process, IMP biosynthetic process, regulation of mRNA splicing *via* spliceosome, and cellular biogenic amine metabolic process. Upregulation was detected on genes encoding proteins like spermidine synthase, diamine acetyltransferase 2, kynurenine formamidase, low-density lipoprotein receptor-related protein 2, and the myomodulin neuropeptide; and downregulation was observed on genes encoding the disks large-associated protein 1, brorin, and the 5-hydroxytryptamine receptor 1 (Table 3).

**Table 3:** Functional enrichment for differentially expressed genes in oviducal glands of *Octopus maya* at each condition.

GO term	FE	*P*-value
MAT24		
fat-soluble vitamin metabolic process	30.28	3.83E−03
IMP biosynthetic process	20.18	8.90E−03
negative regulation of RNA splicing	13.76	4.01E−04
cellular biogenic amine metabolic process	12.75	3.44E−03
cellular amine metabolic process	9.18	1.95E−03
regulation of mRNA splicing, *via* spliceosome	6.49	2.15E−03
extracellular structure organization	4.07	3.32E−03
alpha-amino acid metabolic process	3.41	4.68E−03
nucleotide biosynthetic process	3.32	1.00E−02
DNA replication	3.06	8.77E−03
FER24		
positive regulation of calcineurin-NFAT signaling cascade	31.33	2.96E−03
regulation of male germ cell proliferation	18.80	9.45E−03
positive regulation of cholesterol efflux	18.80	9.45E−03
cell volume homeostasis	13.05	4.04E−04
water homeostasis	12.53	3.23E−03
hydrogen peroxide catabolic process	11.39	4.34E−03
phagocytosis, engulfment	9.64	7.17E−03
protein hydroxylation	9.64	7.17E−03
serine family amino acid biosynthetic process	8.95	8.92E−03
retinoid metabolic process	7.23	1.19E−03
muscle organ morphogenesis	6.53	6.42E−03
cellular hormone metabolic process	4.95	6.65E−03
positive regulation of cell-substrate adhesion	4.70	8.28E−03
regulation of reproductive process	4.14	6.40E−03
cellular amino acid catabolic process	3.86	2.11E−03
extracellular structure organization	3.69	9.70E−05
extracellular matrix organization	3.30	9.64E−04
reactive oxygen species metabolic process	3.03	9.28E−03
glycosylation	2.83	7.98E−04
glycoprotein metabolic process	2.42	1.70E−03
SEN24		
regulation of microvillus organization	58.80	8.40E−04
cellular hormone metabolic process	9.28	4.09E−04
retinoid metabolic process	9.05	9.22E−03
iron ion homeostasis	8.91	2.18E−03
transition metal ion transport	7.06	1.47E−03
hormone metabolic process	5.35	1.84E−03
cellular response to growth factor stimulus	3.27	3.18E−04
lipid metabolic process	1.99	3.69E−03
FER24 *vs* FER-TD		
extracellular matrix disassembly	26.13	7.08E−02
plasma membrane organization	7.56	5.45E−02
regulation of cell adhesion	5.75	8.79E−02
single-organism membrane organization	3.45	9.63E−02
signaling	1.72	7.87E−02

**Note:**

GO, gene ontology; FE, fold enrichment; MAT24, mated; FER24, fertilized; SEN24, senescence; all at 24 °C. FER-TD, fertilized and exposed to the heat shock treatment with temperature decrease.

DEGs obtained for FER24 enriched biological processes like the regulation of calcineurin-NFAT signaling cascade, cellular water homeostasis, regulation of male germ cell proliferation, cellular hormone metabolic process, regulation of reproductive process, extracellular matrix organization, and glycosylation. Upregulation was detected on genes encoding aquaporin, calcium and integrin-binding protein 1, chorion peroxidase, dopamine receptor 1, FMRFamide-activated amiloride-sensitive sodium channel, peroxiredoxin-4, protein catecholamines up, 5-hydroxytryptamine receptor 1, and 16 genes included in the glycoprotein biosynthetic process GO term (Table 4), like the beta-1,4-N-acetylgalactosaminyltransferase bre-4; by contrast, downregulation was observed on genes encoding the 3-oxo-5-alpha-steroid 4-dehydrogenase 1, polypyrimidine tract-binding protein 1, and the myomodulin neuropeptide.

**Table 4:** Transcripts corresponding to the glycoprotein biosynthesis process upregulated in the FER24 condition.

Transcript ID	UniProt ID	Gene name	Protein name
TRINITY_DN43413_c0_g1_i1	ALG8_HUMAN	ALG8	Probable dolichyl pyrophosphate Glc1Man9GlcNAc2 alpha-1,3-glucosyltransferase
TRINITY_DN13830_c0_g1_i5	B3GN5_PIG	B3GNT5	Lactosylceramide 1,3-N-acetyl-beta-D-glucosaminyltransferase
TRINITY_DN13397_c0_g2_i3	BRE4_CAEBR	bre-4	Beta-1,4-N-acetylgalactosaminyltransferase bre-4
TRINITY_DN5237_c0_g1_i1	CANT1_HUMAN	CANT1	Soluble calcium-activated nucleotidase 1
TRINITY_DN16173_c0_g2_i10	D19L1_HUMAN	DPY19L1	Probable C-mannosyltransferase DPY19L1
TRINITY_DN16072_c0_g1_i2	EDEM2_HUMAN	EDEM2	ER degradation-enhancing alpha-mannosidase-like protein 2
TRINITY_DN16442_c0_g2_i1	FUCTA_DROME	FucTA	Glycoprotein 3-alpha-L-fucosyltransferase A
TRINITY_DN14002_c0_g1_i1	G3ST2_MOUSE	Gal3st2	Galactose-3-O-sulfotransferase 2
TRINITY_DN11335_c0_g1_i1	GALT9_CAEEL	gly-9	Probable N-acetylgalactosaminyltransferase 9
TRINITY_DN3280_c0_g1_i1	GCNT1_MOUSE	Gcnt1	Beta-1,3-galactosyl-O-glycosyl-glycoprotein beta-1,6-N-acetylglucosaminyltransferase
TRINITY_DN10779_c0_g1_i1	GOGA2_RAT	Golga2	Golgin subfamily A member 2
TRINITY_DN16242_c0_g1_i8	LRP2_HUMAN	LRP2	Low-density lipoprotein receptor-related protein 2
TRINITY_DN29933_c0_g1_i1	MGT4B_DANRE	mgat4b	Alpha-1,3-mannosyl-glycoprotein 4-beta-N-acetylglucosaminyltransferase B
TRINITY_DN11201_c0_g1_i1	PMGT1_HUMAN	POMGNT1	Protein O-linked-mannose beta-1,2-N-acetylglucosaminyltransferase 1
TRINITY_DN10762_c0_g1_i1	STT3A_BOVIN	STT3A	Dolichyl-diphosphooligosaccharide--protein glycosyltransferase subunit STT3A
TRINITY_DN7997_c0_g1_i1	TMM59_MOUSE	Tmem59	Transmembrane protein 59

**Note:**

These transcripts were upregulated in oviducal glands of fertilized *Octopus maya* females in the Control condition (24 °C).

**Figure 3:**
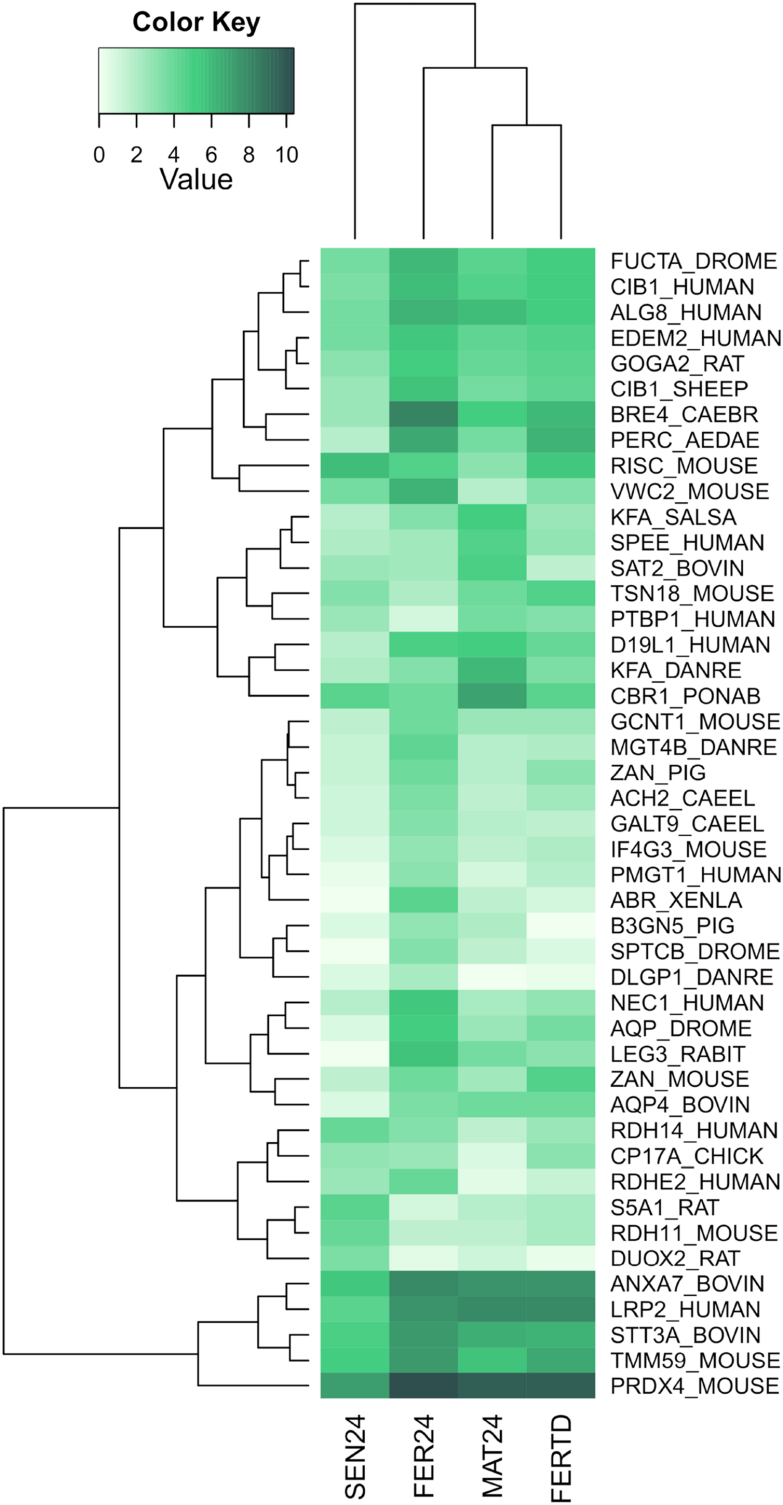
Heatmap of top DEGs among the physiological stages of oviducal glands of *O. maya*. Row labels indicate the UniProt IDs of the best BLASTx hit for each transcript. MAT24 for mated females at 24 °C, FER24 for fertilization in females at 24 °C, SEN24 for senescent females at 24 °C and, FERTD for fertilization in females from the HTST-TD. Values in log2(TPM+1).

In SEN24, DEGs enriched biological processes like the cellular hormone metabolic process, iron ion homeostasis, cellular response to growth factor stimulus, lipid metabolic process, and oxidation-reduction process. Upregulation was observed on the retinoid-inducible serine carboxypeptidase, retinol dehydrogenase 14, 3-oxo-5-alpha-steroid 4-dehydrogenase 1, and matrix metalloproteinase-19; while downregulation was observed on genes encoding the chorion peroxidase, galectin-3, protein catecholamines up, FMRFamide-activated amiloride-sensitive sodium channel, and the cAMP-responsive element modulator. The top differentially expressed genes (DEGs) among the different physiological stages were plotted and clustered in a heatmap (Fig. 3). In this heatmap, the conditions MAT24 and FER-TD were grouped due to similar gene expression patterns.

**Figure 4:**
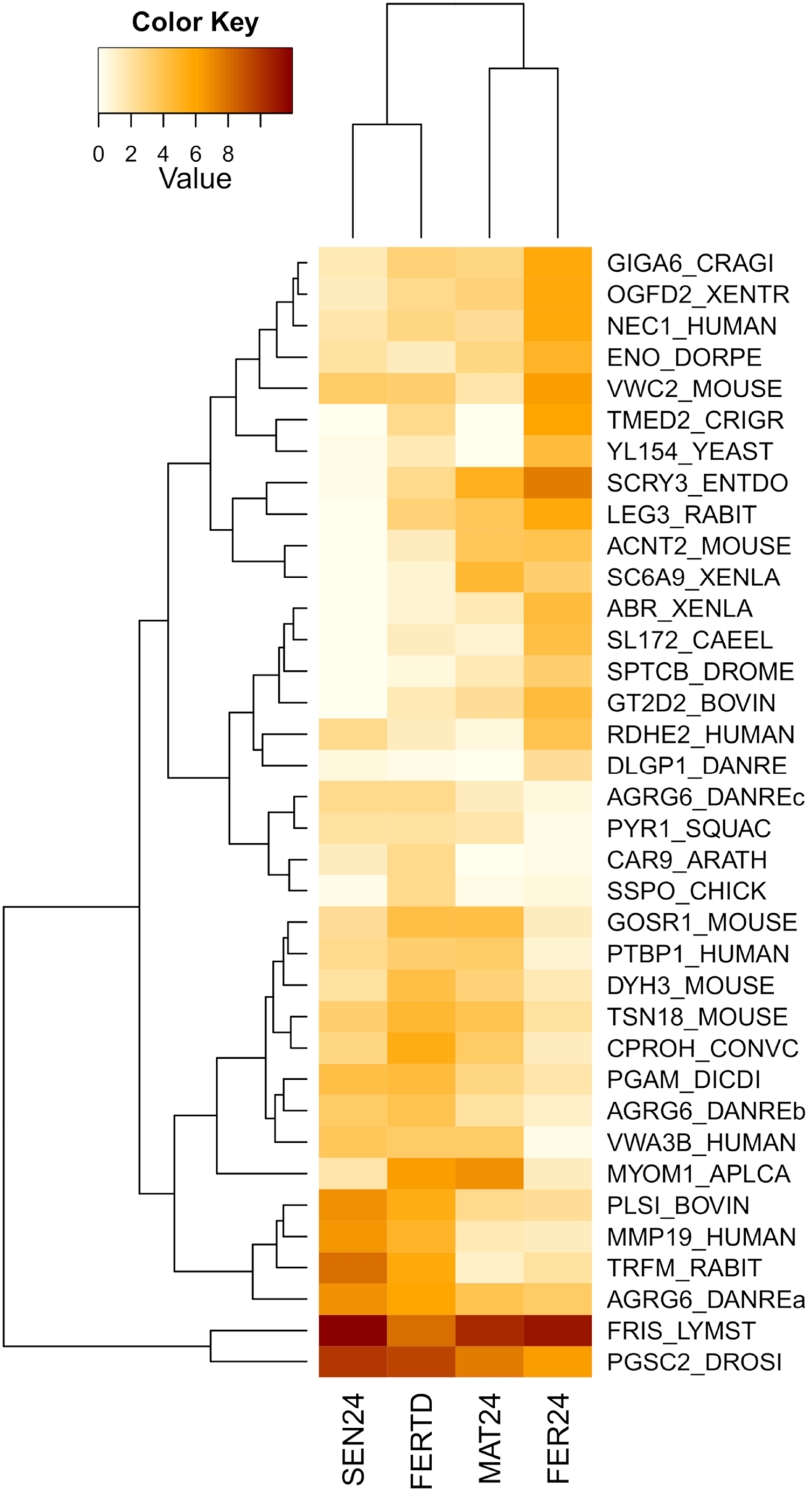
Heatmap of top DEGs from the comparison between *O. maya* oviducal gland samples of the Control condition against samples of the HTST-TD. Row labels indicate the UniProt IDs of the best BLASTx hit for each transcript. MAT24 for mated females at 24 °C, FER24 for fertilization in females at 24 °C, SEN24 for senescent females at 24 °C and, FERTD for fertilization in females from the HTST-TD. Values in log2(TPM+1).

In the comparison between FER24 *vs* FER-TD, DEGs enriched biological processes like the extracellular matrix disassembly, plasma membrane organization, regulation of cell adhesion, signaling, and cell communication. In FER-TD, we detected upregulation on genes encoding the CAD protein, matrix metalloproteinase-19, myomodulin neuropeptide, and tetraspanin-18; whereas strong downregulation was detected on the gene encoding the lactosylceramide 1,3-N-acetyl-beta-D-glucosaminyltransferase. By contrast, FER24 showed higher expression of genes encoding brorin, galectin-3, epidermal retinol dehydrogenase 2, neuroendocrine convertase 1, and spectrin beta chain. The top DEGs between the Control and the HTST-TD conditions were plotted and clustered in a heatmap (Fig. 4). In this heatmap, the conditions SEN24 and FER-TD showed similar expression patterns and were grouped, another group consisted of MAT24 and FER24.

**Figure 5:**
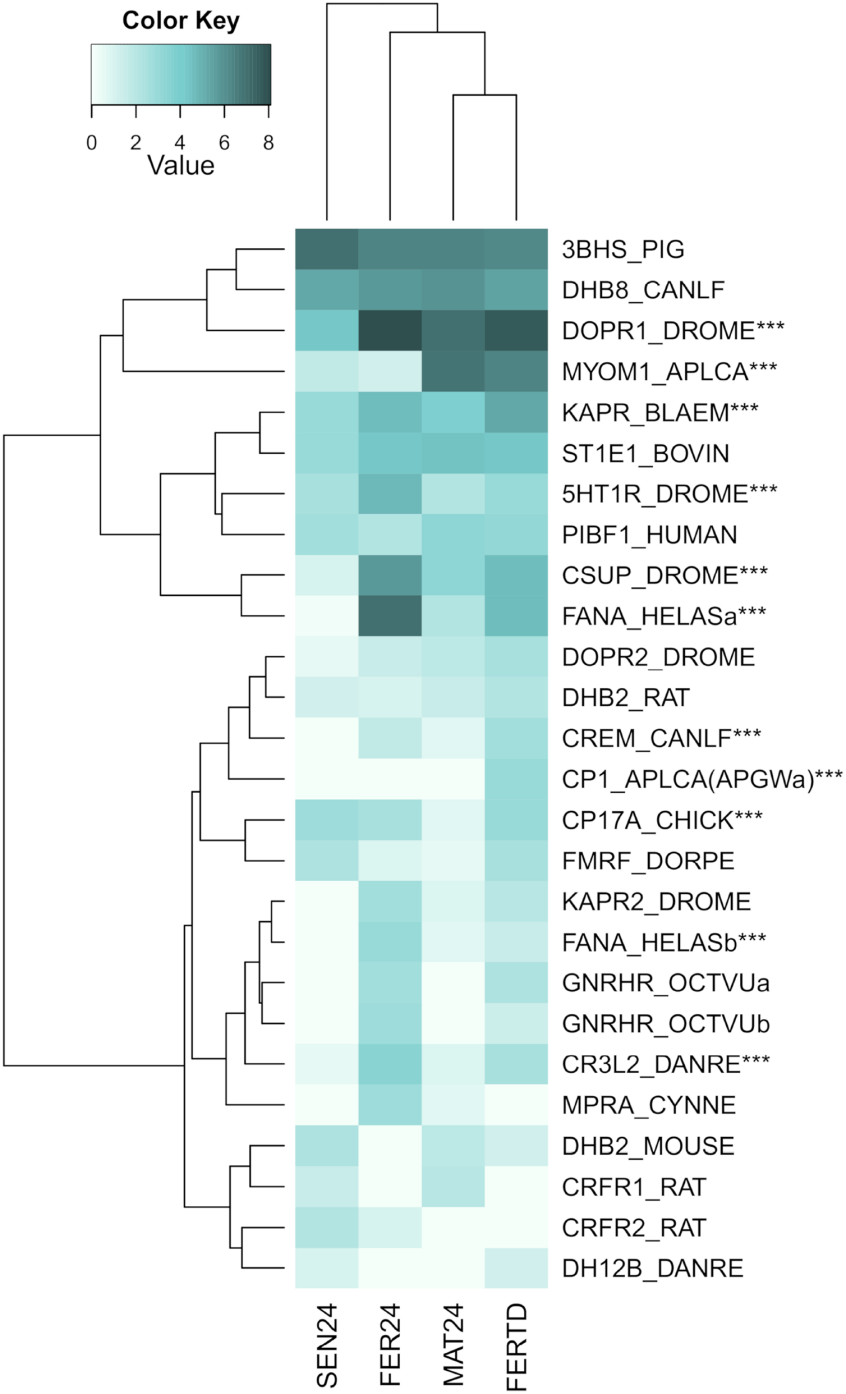
Expression heatmap for regulatory genes of oviducal glands’ activity in *O. maya* at different conditions. Row labels indicate the UniProt IDs of the best BLASTx hit for each transcript. MAT24 for mated females at 24 °C, FER24 for fertilization in females at 24 °C, SEN24 for senescent females at 24 °C and, FERTD for fertilization in females from the HTST-TD. Values in log2(TPM+1), ***DEGs (fold change > 2, false discovery rate < 0.05).

A third heatmap was constructed for transcripts related to the regulation of the OvG activity (Fig. 5). In this heatmap, stronger expression differences were evident in genes encoding the 5-hydroxytryptamine receptor 1 (5HT-7), cAMP-dependent protein kinase regulatory subunit (PKAR), cAMP-responsive element modulator (CREM), dopamine receptor 1 (Dop1R1), FMRFamide-activated amiloride-sensitive sodium channel (FanaCh), protein catecholamines up (Catsup), and steroid 17-alpha-hydroxylase/17,20 lyase (CYP17A1) showing all an expression peak at the fertilization stage (FER24); and myomodulin (MYOM) which was upregulated in MAT24 and FER-TD. In this heatmap, the conditions MAT24 and FER-TD were grouped due to similar expression patterns. Annotations of upregulated transcripts in each reproductive stage are shown in Table S1. The list of all transcripts used in heatmaps including their gene products is shown in Table S2.

### Validation of gene expression by RT-qPCR

Three different gene expression patterns were represented: the upregulation in MAT24 was represented by the genes MYOM and SAT2; the upregulation in FER24 by Ptx, Dop1R1, PRDX4, and Catsup; and the upregulation in SEN24 by the genes MMP-19 and SRD5A1. Primer efficiency ranged from 92.1 to 109.8, the annealing temperatures, product sizes, and sequences of each primer pair are shown in Table 5. The elongation factor 1 beta (EF1B) and the ribosomal protein L6 (RPL6) showed higher stability and were used as reference genes. Gene expression values estimated by RNA-Seq and RT-qPCR methods showed a significant Spearman correlation (*P*-value = 0.009). A scatter plot with linear regression representing the correlation between RNA-Seq and RT-qPCR gene expression values is provided as Fig. S1.

**Table 5:** Primers for target and reference genes of *Octopus maya* oviducal glands used in RT-qPCR.

Transcript	Protein name	E	Tm	Size	Primers
TRINITY_DN22438_c0_g1_i1	3-oxo-5-alpha-steroid 4-dehydrogenase 1	109.8	60	119	CGGAAACCTAACGAAACAGG
					GACCAGCATGCAATAGCAAA
TRINITY_DN16362_c4_g6_i1	Chorion peroxidase	97.3	60	116	CGGCTTATCACGACGGTTAT
					GCATTTTGCGTTGAAAGGTT
TRINITY_DN14910_c0_g1_i2	Diamine acetyltransferase 2	90.5	60	127	ACCCACCTTCTGTTGATGATCT
					ACCAATGGTCCTGTGCTTAGT
TRINITY_DN12903_c1_g1_i1	Dopamine receptor 1	91.4	60	120	GGCTGTGACCTCGACATCAA
					GTGTTGCTGAGCCGTACTGT
TRINITY_DN12984_c0_g1_i1	Matrix metalloproteinase-19	94	60	150	TGACGAAGAATGGACTGCAA
					TGGAAATCCTTCACGAAACC
TRINITY_DN32740_c0_g1_i1	Myomodulin neuropeptides 1	97.2	62	118	GCAGTGGACCATTCCTTGAT
					TTTTCGAAGCCATTTTGTCC
TRINITY_DN16150_c1_g1_i4	Peroxiredoxin-4	100.9	60	107	ATGGCCAAGATTCTGAAGGA
					CACCACGAAACAAAGGAGGT
TRINITY_DN15757_c0_g1_i2	Protein catecholamines up	98.4	60	114	TTGGGTCTGCGAGTCTTCTT
					AGCCATTCTCACAGCGAAGT
Reference (Xu & Zheng, 2018)	60S ribosomal protein L6	92.1	60	171	GGAAGGCACAAGGGAAAGCG
					CCTGGCTGGGATCTGAACCT
Reference (Juárez, 2016)	Elongation factor 1B	95.3	60	108	TGATGTCAAACCATGGGACG
					AGAGGTGCTAACTTGGACGC

**Note:**

E, primer efficiency; Tm, annealing temperature; Size, product size in base pairs.

**Figure 6:**
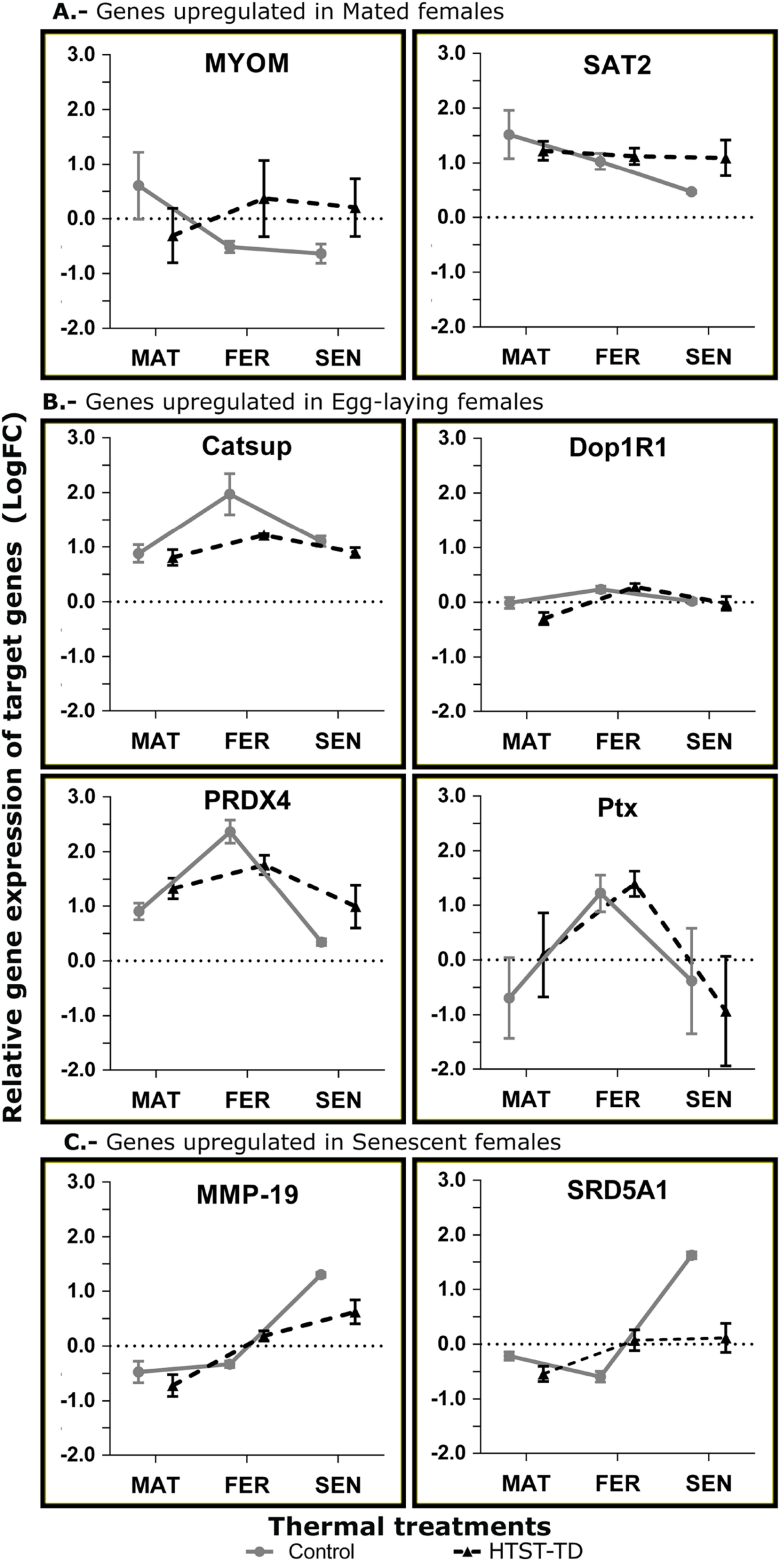
Relative expression of target genes (RT-qPCR, average and standard deviation) at different conditions of oviducal glands of *O. maya*. Comparison between the Control temperature (24 °C) and HTST-TD. MAT, mated; FER, fertilization; SEN, senescence. Relative expression (fold change, FC) was calculated using the 60S ribosomal protein L6 and Elongation factor 1 B as reference genes (values in Log10 scale). The expected gene expression patterns (A, B, and C) were estimated in the Control condition by bioinformatic methods.

In the RT-qPCR estimation, the gene SAT2 showed a higher expression in the mated stage (MAT24) but the differences were not significant. The MYOM gene showed a significant effect for the interaction of factors (*P* = 0.0304) with higher expression in MAT24 and FER-TD (Fig. 6A). The genes with an expected peak in the fertilization stage showed significant effect for the “stage” factor (Catsup, *P* = 0.0029; Dop1R1, *P* = 0.0064; Ptx, *P* = 0.0161; PRDX4, *P* = 0.0002) with higher expression in fertilized females. However, the gene Catsup also showed a significant effect of the “treatment” factor (*P* = 0.0219) with higher expression in the Control (Fig. 6B). The genes with a bioinformatic peak in the senescence stage (SEN24) MMP-19 and SRD5A1 showed in the RT-qPCR a significant effect for the “stage” factor with higher expression in senescent females (both *P* < 0.0001); they also showed significant effect due to the interaction of factors (*P* = 0.0284 and 0.0001 respectively), and SRD5A1 showed significant effect due to the “treatment” factor (*P* = 0.0104) with the highest expression in SEN24 (Fig. 6C).

## Discussion

### Mated stage

Before the fertilization, the spermatozoa stored in the OvG must be reactivated (De Lisa et al., 2013). Important genes involved in the axoneme assembly and cilium motility such as SPEF2, TTLL1, TTLL3, DRC1, CCDC39, RSPH4A, DNAH1, SPAG16, and LRGUK were detected in the OvG transcriptome. Mutations in these genes have been associated with sperm immobility and male infertility, which suggests that they are essential for fertilization (Leigh et al., 2009; Berg et al., 2011; Onoufriadis et al., 2014; Pereira et al., 2017; Wu et al., 2016; zur Lage, Newton & Jarman, 2019; Takeuchi et al., 2020). The expression of this set of genes in the OvG suggests that spermatozoa are stored in spermathecae without functional tails or disassembled and that flagellar components involved in sperm motility are reassembled there just before fertilization. Interestingly, in the spermathecae epithelium, spermatozoa are immobilized with their heads inserted within the mucosa, while the tails are not visible (Di Cosmo, Di Cristo & Paolucci, 2001; Olivares et al., 2017).

Females of *Octopus vulgaris* can store sperm in their OvG for up to 10 months (Mangold, 1987), which implies that during this prolonged period, the spermatozoa integrity depends on the protective molecular mechanisms of the female. For example, the upregulation of the AFMID gene, encoding the kynurenine formamidase, in mated females of *O. maya* may contribute to the protection of spermatozoa since it is important to eliminate toxic metabolites (Dobrovolsky et al., 2005).

In mated *O. maya* females, genes associated with the synthesis of the biogenic amines spermine and spermidine like SAT2 and SRM (Chen et al., 2003; Wu et al., 2007) were upregulated. In mammals, spermine and spermidine play a protective role for spermatozoa since they inhibit the glycation and fragmentation of sperm DNA in the epididymis (Méndez & Sauer-Ramírez, 2018). These biogenic amines also have a key role in the precise timing for successful fertilization; seminal spermine prevents premature capacitation and acrosome reaction (Rubinstein & Breitbart, 1991). Moreover, *in vitro* studies showed that spermine enhances the activity of seminal maltase, which increases glucose utilization by spermatozoa (Sheth & Moodbidri, 1977). High spermine and spermidine levels may be required in the OvG of mated females to maintain the integrity of spermatozoa and to inhibit fertilization until it is induced by environmental and metabolic signals. In this species, such a mechanism could guarantee a precise temporality for sperm activation and fertilization of eggs (Wells & Wells, 1959; O’Dor & Wells, 1978; Di Cristo, 2013). Interestingly, genes encoding the 5-hydroxytryptamine receptor 1 (serotonin receptor) and the gonadotropin-releasing hormone receptor (GnRHR) showed a lower expression at the mated stage (MAT24), suggesting that these receptors are not required before fertilization in the OvG under optimal thermal conditions.

The lifecycle of octopuses is characterized by a physiological transition from the growth to the reproductive phase: after reaching enough energy reserves during the growth phase, the female stops feeding and dedicates exclusively to egg-laying and egg-care (O’Dor & Wells, 1978; Di Cristo, 2013). Such transition seems to be mediated by regulatory neuropeptides and catecholamines (Di Cristo, 2013; Wang & Ragsdale, 2018). These regulatory peptides are typically abundant in non-mated (growing) individuals which display hunting and active feeding (Wang & Ragsdale, 2018). Interestingly, the high expression of specific neuropeptides opposes certain reproductive events. In the OvG of *O. maya*, the myomodulin neuropeptide was upregulated in the MAT24 condition. The RT-qPCR estimation confirmed that, in optimal thermal conditions, a decrease in the expression of the MYOM gene coincided with the onset of fertilization. Myomodulin has been detected in mollusk of genera like *Aplysia* (Cropper et al., 1987, 1991; Brezina et al., 1995), *Lymnea* (Kellett et al., 1996), *Haliotis* (York et al., 2012), *Helix* (Greenberg et al., 1997), and *Sepia* (Zatylny-Gaudin et al., 2016). This neuropeptide has been associated with the modulation of feeding rates and muscle contractions (Cropper et al., 1987, 1991; Brezina et al., 1995; Kellett et al., 1996; Greenberg et al., 1997). More recently, a role of myomodulin in the regulation of egg-laying was suggested, since it was detected in the OvG of *Sepia officinalis* (Zatylny-Gaudin et al., 2016) and showed an upregulation before the spawning in *Haliotis asinina* females (York et al., 2012), which coincides with was found in the present study. Structurally, the oviducal glands consist of mixed layers of muscular and connective tissue vascularized and innervated (Peterson, 1959; Budelmann, Schipp & von Boletzky, 1997; Olivares et al., 2017; Anadón, 2019). Myomodulin is an important regulatory neuropeptide in the multi-messenger innervation of the sexual organs with muscle tissue in mollusks, including the glands (De Lange, Joosse & Van Minnen, 1998; Koene, 2010). Thus, it is possible that during the mated stage the OvG has greater vascularization and innervation, and therefore a higher expression of myomodulin, as was observed.

### Fertilization stage

Downregulation of the myomodulin gene was observed in FER24 and strengthens the idea that the downregulation of some neuropeptides precedes reproductive events in *Octopus* (Wang & Ragsdale, 2018). In the OvG of *O. maya*, it seems that myomodulin must be downregulated for the onset of fertilization.

In the *O. vulgaris* female, fertilization is partially controlled by steroid hormones like progesterone and 17β-estradiol, whose levels fluctuate through the reproductive phase (Di Cosmo et al., 1998; Di Cosmo, Di Cristo & Paolucci, 2001; Tosti et al., 2001). These hormones are associated with the growth and differentiation of the reproductive system, including the OvG, and the remobilization of spermatozoa (Di Cosmo, Di Cristo & Paolucci, 2001; Tosti et al., 2001). In the present study, a high expression of the Mpra gene was detected in the OvG of fertilized females (FER24); this gene encodes the membrane progestin receptor alpha, which binds to progesterone (Zhu et al., 2003); this coincides with was found in *O. vulgaris* (Di Cosmo et al., 1998) and suggests that the role of sex steroids in the control of reproduction could be a more generalized adaptation among octopus species. Another endocrine gene upregulated in the fertilization stage was the CYP17A1, encoding the steroid 17-alpha-hydroxylase/17,20 lyase, which participates in the metabolism and synthesis of steroid hormones (Auchus, Lee & Miller, 1998; Strauss, Modi & McAllister, 2014; Petrunak et al., 2014; Yoshimoto et al., 2016). These results suggest that OvGs metabolize and secrete steroid hormones to coordinate the activity of different organs for successful fertilization and egg-laying. Alternatively, Di Cristo & Di Cosmo (2007) proposed that sex steroids in the OvG may play a role in sustaining the production and secretion of the mucoproteins and mucopolysaccharides that coat the eggs. Authors also suggested that cyclic AMP (cAMP) modulates the secretory activity of the OvG (Di Cristo & Di Cosmo, 2007). In the present study, we detected upregulation on genes encoding the cAMP-dependent protein kinase regulatory subunit (PKAR) and cAMP-responsive element modulator (CREM) at the fertilization stage. This suggests a high secretory activity of the OvG during the fertilization stage. Moreover, the CREM gene has been associated with male fertility (Pati, Meistrich & Plon, 1999; Yanagimachi et al., 2004).

The expression of putative sperm genes was conspicuous during the fertilization stage. For instance, the Catsup gene encoding the protein catecholamines-up showed an upregulation in FER24. This protein is a zinc ion transmembrane transporter; involved in the zinc ion influx required for sperm capacitation and fertilization in mammals (Kerns, Zigo & Sutovsky, 2018; Kerns et al., 2018). Likewise, CIB1 and PRDX4 which are key genes for male fertility (Yuan et al., 2006; Iuchi et al., 2009), were upregulated at the fertilization stage. Furthermore, transcripts of the Drip gene, encoding aquaporin also showed upregulation in FER24. It is well known that aquaporins are essential for male fertility since they are responsible for the regulation of sperm volume, which is crucial for fertilization. They are also important for the osmotic adaptation of the spermatozoa to different microenvironments until reaching the egg, and for maintaining the osmotic homeostasis for both male and female gametes during fertilization (Delgado-Bermúdez, Ribas-Maynou & Yeste, 2022; Yeung et al., 2009; Ribeiro et al., 2021). The high expression of sperm genes during the fertilization stage is evidence of the synchronized reactivation of the sperm that was stored in the spermathecae.

A key step in fertilization (internal or external) is the interaction between male and female gametes (*i.e*., gametic compatibility). The union of the spermatozoon to the extracellular matrix of the egg is mediated by gamete recognition proteins (GRPs) which have an important influence on the reproductive success of taxa with external or internal fertilization (Panhuis, Clark & Swanson, 2006; Kosman & Levitan, 2014). In our study, the ZAN gene encoding zonadhesin showed upregulation in the fertilization stage. Zonadhesin is a sperm protein that binds to the pellucid zone in a species-specific manner in mammals (Hardy & Garbers, 1995; Gao & Garbers, 1998; Bi et al., 2003; Tardif et al., 2010; Springate & Frasier, 2017). At the same time, LGALS3 which encodes galectin-3 was upregulated. Galectin-3 is a lectin that also participates in the binding of spermatozoa to the pellucid zone (Mei et al., 2019); therefore, in an analogous approach, ZAN and LGALS3 may cooperate in the union of male and female gametes in this octopus species.

After the fertilization, the egg envelope (chorion) hardens (Wells, 1978); this process is crucial to block polyspermy in internal fertilization, protecting the embryo from mechanical damage and preventing bacterial infections (Wang et al., 2021). The hardening of the egg envelope requires the activity of the chorion peroxidase, encoded by the Ptx gene. This peroxidase is essential for the cross-link of chorion proteins and participates in chorion melanization (Mindrinos et al., 1980; Margaritis, 1985; Li, Hodgeman & Christensen, 1996; Han, Li & Li, 2000; Konstandi et al., 2005, 2006; Li & Li, 2006; Wang et al., 2021). In the present study, the Ptx gene peaked at the fertilization stage of *O. maya*.

Later, eggs are coated with a slime that is composed of mucoproteins secreted from the peripheral gland and a sulfonated mucopolysaccharide from the central gland (Froesch & Marthy, 1975). Mucoproteins are heavily glycosylated containing *O*-linked oligosaccharide chains that are covalently attached to serine or threonine residues of their polypeptide backbones (Brockhausen & Stanley, 2015). In these proteins, glycosylation begins with the addition of N-acetylgalactosamine by a large family of UDP-GalNac:polypeptide N-acetylgalactosaminyltransferases (Clausen & Bennett, 1996). In the present study, it was detected a set of 16 genes involved in the glycoprotein biosynthetic process and glycosylation process (ALG8, B3GNT5, bre-4, CANT1, DPY19L1, EDEM2, FucTA, Gal3st2, gly-9, Gcnt1, Golga2, LRP2, mgat4b, POMGNT1, STT3A, and Tmem59) including N-acetylgalactosaminyltransferases, showing upregulation in FER24. Therefore, this set of genes may be crucial for slime synthesis and important for normal egg-laying in *O. maya*.

Finally, the eggs enter the distal oviduct and are transported by peristalsis, one behind the other. In the Control condition, the receptors of serotonin (5HT-7), and dopamine (Dop1R1) were upregulated during the egg-laying in the OvG of *O. maya* females. Interestingly, serotonin and dopamine were identified as stimulating neurotransmitters that induce spawning in bivalves (Arendse, Pitcher & Griffiths, 2018; Gibbons & Castagna, 1984; Braley, 1985; Osada, Matsutani & Nomura, 1987; Deguchi & Osada, 2020). In this sense, the high expression of these receptors at the fertilization stage suggests that serotonin and dopamine are required to induce egg-laying also in *O. maya* females. Clusters of eggs and slime are then released; the female molds an egg string and fixes the end of the string to a suitable substrate (Froesch & Marthy, 1975).

### Senescence stage

Once fertilization and egg-laying were finalized, there was a downregulation of important reproductive genes mentioned above, including Ptx, Catsup, FanaCh, CREM, and LGALS3. On the other hand, one of the genes with the highest expression in SEN24 was the SRD5A1. This gene encodes the 3-oxo-5-alpha-steroid 4-dehydrogenase 1, which participates in the metabolism of steroid hormones like progesterone. The expression of SRD5A1 highly increased from the fertilization to senescence stage suggesting that fertilization and egg-laying may terminate due to an enzymatic depletion of progesterone. On the other hand, this enzyme participates in the synthesis of allopregnanolone, a neuroactive metabolite of progesterone that acts in the brain (Tsutsui & Haraguchi, 2016). In senescent *O. maya* females, the OvGs may release this steroid into the bloodstream to reach the central nervous system.

Another gene highly expressed in senescent females was the MMP-19 encoding the matrix metalloproteinase-19, which participates in the degradation of the extracellular matrix. This gene has been associated with wound healing and tissue remodeling (Nagase & Murphy, 2013), which may be part of the natural senescence process. In senescent females of *O. vulgaris*, *O. mimus*, and *O. maya* (present study), the OvG notably shrank compared to the previous fertilization stage (Cuccu et al., 2013; Olivares et al., 2017), which may imply a tissue remodeling process.

### Effect of high temperatures on fertilization stage

FER-TD and MAT24 were grouped in the cluster analyses for DEGs among the stages, and a set of regulatory genes. In these genes, the expression levels in FER-TD resemble those of the previous stage (MAT24) of the Control, suggesting that certain processes were delayed due to the HTST-TD. This supports the idea that octopus females under thermal stress can delay certain reproductive processes until temperatures become favorable (Juárez et al., 2015).

In the cluster analysis of DEGs between the Control and the HTST-TD, which are those directly affected by the thermal stress, FER-TD was grouped with the senescence condition SEN24 of the Control. This grouping may be related to a high physiological deterioration of the gland in FER-TD, which resembles that of the senescence stage. There was no grouping between FER24 and FER-TD in any dendrogram, confirming that thermal stress drastically affected gene expression in the oviducal glands during the fertilization stage.

In *O. maya*, the temperature is an important modulator of fertilization and egg-laying rates. These rates are significantly reduced by temperatures above 27 °C (Juárez et al., 2015), and in the males, temperatures around 30 °C provoke damages in testis and spermatozoa (López-Galindo et al., 2019a, 2019b). Gene expression changes in the OvG caused by temperature may be the cause of the reduced fertilization and egg-laying rates observed in this species.

Previously, we discussed the inhibitory role for fertilization of the myomodulin neuropeptide in MAT24, and that the MYOM gene must be downregulated to initiate fertilization. However, under thermal stress this gene was not downregulated during the fertilization stage, instead, it was highly expressed. In gastropods, this bioactive neuropeptide potentiates muscular contractions (Cropper et al., 1987; Kellett et al., 1996; Greenberg et al., 1997). Similarly, the gene encoding the APGW-amide neuropeptide (cerebral peptide 1) significantly increased its expression under thermal stress. This neuropeptide also potentiates muscle contractions in bivalves and gastropods (Minakata et al., 1991; Henry, Zatylny & Favrel, 2000), and in the OvG of *O. vulgaris*, where a function in the oviduct contractility was proposed (Di Cristo & Di Cosmo, 2007). Therefore, we can hypothesize that in *O. maya* the coordinated release of myomodulin and APGW-amide may keep the proximal oviduct contracted for longer periods, limiting the passage of eggs from the ovary to the OvG, thus reducing the fertilization rate under high temperatures as was observed in this octopus species (Juárez et al., 2015). This hypothesis should be evaluated in future research.

Essential genes for successful fertilization were downregulated in the HTST-TD; for instance, the gene LGALS3, which participates in the union of the spermatozoon to the extracellular matrix of the egg (Mei et al., 2019) showed high levels in the Control condition but was downregulated in the HTST-TD. Similarly, the gene VWC2, encoding the brorin protein, was highly expressed in the optimal temperature and downregulated in the HTST-TD; brorin is also involved in cell adhesion (Manabe et al., 2008).

Although the activity of OvG is partially regulated by steroid hormones (Di Cosmo et al., 1998; Di Cosmo, Di Cristo & Paolucci, 2001), our results suggest that these glands also play a role in the biosynthesis and release of hormones. In this regard, under the optimal temperature, an upregulation of the Pcsk1 gene was detected, while it was downregulated in the HTST-TD. This gene encodes the neuroendocrine convertase 1 and is involved in the conversion of secretory precursor proteins to bioactive polypeptides (Morash, Soanes & Anini, 2011). According to our results, the processing of prohormones in the OvG of *O. maya* females is related to fertilization, and the downregulation of the Pcsk1 gene could modulate this process under thermal stress. Another gene with strong downregulation in FER-TD was the B3GNT5, which encodes the Lactosylceramide 1,3-N-acetyl-beta-D-glucosaminyltransferase. In mice, the knock-out of this gene provokes a series of reproductive defects, therefore it is a key gene for successful reproduction (Kuan et al., 2010).

### Ecological implications of a temperature-driven inhibition of fertilization in *O. maya*

Elevated temperatures caused gene expression changes in the oviducal gland that may be associated with the low fertilization and egg-laying rates observed in this octopus species (Juárez et al., 2015). The negative regulation of fertilization rates under elevated temperatures may be a strategy that prevents excessive thermal stress for embryos and hatchlings, which can improve populations’ fitness (Juárez et al., 2015, 2016; Caamal-Monsreal et al., 2016); but at the same time, such a mechanism makes this species vulnerable to ocean warming and interannual thermal anomalies. Stational upwelling pulses modulate the thermal conditions at the Northeast region of the peninsula, maintaining suitable temperatures for octopus reproduction all year long, but its influence weakens towards the Western region, where sea temperature rises especially during thermal anomaly events (Angeles-Gonzalez et al., 2017). The incidence of thermal anomalies has been associated with a decrease in yields of the octopus’ fishery in the Yucatan Peninsula, especially in the Western region (Noyola Regil et al., 2015; Angeles-Gonzalez et al., 2017). In an ocean warming scenario or during thermal anomalies, octopuses from Western Yucatan Peninsula may move away into deeper environments or towards the upwelling zone, looking for cooler waters. This may alter ecological interactions and increase the mortality rate if the species finds additional predators or competitors while reaching such environments. In this scenario, the *O. maya* fishery may decline due to a reduction in fertilization and egg-laying rates, but also because the population could migrate to deeper waters reducing the species catchability, or because of a higher mortality rate (Ángeles-González et al., 2021). In this regard, the octopus aquaculture—with stringent temperature control—can emerge as the best alternative for octopus’ production.

### Limitations of the study and recommendations for further research

In the sequencing step, it is recommended to use at least three biological replicates per condition (or more, if the funding allows it) to enhance the statistical power of the analysis, especially to detect DEGs with low expression (Williams et al., 2014; Honaas, Altman & Krzywinski, 2016). However, due to funding constraints, we implemented the biological averaging approach by using pooled samples per condition in the sequencing step (Honaas, Altman & Krzywinski, 2016) and utilized biological replicates to validate the expression of key genes through RT-qPCR analysis. This low-cost strategy provided valuable insights into the physiology and thermal stress response in the oviducal gland of *O. maya*.

On the other hand, considering that the oviducal gland consists of multiple sections with specialized tissues (Froesch & Marthy, 1975; Olivares et al., 2017; Anadón, 2019), we recommend, in further research, the use of single-cell transcriptomics to better understand the role of each section in the glands’ physiology.

## Conclusions

At optimal temperatures, key reproductive genes in the OvG control the onset of fertilization and egg-laying: Before fertilization, the upregulation of genes encoding the myomodulin neuropeptide and enzymes for the synthesis of spermine and spermidine may prevent premature fertilization. In the OvG of mated and fertilized females, upregulation of genes related to the assembly and motility of the spermatozoa flagellum indicates the metabolic and transcriptomic reactivation of sperm. During fertilization, genes encoding the receptors of serotonin, dopamine, and progesterone were highly expressed. Likewise, fertilization was favored by the expression of genes that play a role in the interaction of male and female gametes. Other important genes for the reproductive process like Ptx, Catsup, FanaCh, and CYP171A, and those involved in the synthesis of eggshell mucoproteins were conspicuous at this stage under optimal thermal conditions. In senescent females, genes involved in fertilization were downregulated and those involved in the metabolism of steroid hormones like the SRD5A1 were highly expressed.

The fertilization rate decreased in the HTST-TD; this coincided with the upregulation of neuropeptides like myomodulin and APGW-amide, downregulation of genes involved in the adhesion between spermatozoa and eggs like galectin-3 and brorin, and downregulation of the neuroendocrine convertase 1 gene. This regulation may be required to reduce the fertilization rate under high temperatures which are stressful for embryos and hatchlings of this species, therefore the species may be highly vulnerable to ocean warming.

Finally, some proteins associated with the fertilization process in *O. maya* have not been previously detected in other invertebrates, to our knowledge. Although, they have been found in vertebrate taxa, where they exhibit homologies in the molecular mechanisms to achieve fertilization and leave offspring. The reproductive proteins involved in the fertilization process evolve rapidly, partly due to the type of fertilization (internal or external) and the reproductive system (see Table 1 in Turner & Hoekstra (2008)). Thus, the adaptive evolution of functionally reproductive proteins is contrasting between the external fertilization species (mediated by simple gamete proteins) and internal fertilization species (involving complex interactions between multiple proteins) (Turner & Hoekstra, 2008).

## Supplemental Information

10.7717/peerj.12895/supp-1 Supplemental Information 1 Biological processes enriched by differentially expressed genes at each physiological stage of *Octopus maya* oviducal glands. UniProt IDs of the gene products are shown within each gene ontology (GO) term. MAT: mated, FER: fertilization, SEN: senescence, 24: control treatment at 24 °C, TD: heat-shock treatment with temperature decrease.

10.7717/peerj.12895/supp-2 Supplemental Information 2 Transcripts from the oviducal gland transcriptome of *O. maya* selected for heatmap and cluster analysis. E-values belong to the BLASTx searches.

10.7717/peerj.12895/supp-3 Supplemental Information 3 Scatter plot with linear regression for RNA-seq (x) vs RT-qPCR (y) relative gene expression values (in Log2) in oviducal gland samples of *O. maya*. The Spearman correlation for both variables was significant (*P* = 0.009).

10.7717/peerj.12895/supp-4 Supplemental Information 4 ARRIVE guidelines.

10.7717/peerj.12895/supp-5 Supplemental Information 5 RT-qPCR raw data.

## Additional Information and Declarations

### DNA Deposition

The following information was supplied regarding the deposition of DNA sequences: The transcriptome assembly is available at DDBJ/EMBL/GenBank: GJEO00000000.1.

### Data Availability

The following information was supplied regarding data availability: The RT-qPCR raw data is available in the Supplementary File. The raw sequence reads are available at NCBI-SRA: SRR13512014–SRR13512017, BioProject PRJNA544090.
