# Supplementary material for: Oviducal gland transcriptomics of Octopus maya through physiological stages and the negative effects of temperature on fertilization
Source: PeerJ. 2022 Mar 30;10:e12895. doi: 10.7717/peerj.12895 (PMC8976471; doi:10.7717/peerj.12895)
Supplement: Supplemental Information 1 — UniProt IDs of the gene products are shown within each gene ontology (GO) term. MAT: mated, FER: fertilization, SEN: senescence, 24: control treatment at 24 °C, TD: heat-shock treatment with temperature decrease. [file peerj-10-12895-s001.docx]

Supplementary Table S1. Biological processes enriched by differentially expressed genes at each physiological stage of *Octopus maya* oviducal glands.

| **GO term** | **UniProt IDs** |
| --- | --- |
| **MAT24 upregulated** | |
| fat-soluble vitamin metabolic process | AL1A2_HUMAN, CBR1_PONAB, LRP2_HUMAN |
| IMP biosynthetic process | PUR9_CHICK, PUR6_DROME, PUR4_HUMAN |
| negative regulation of RNA splicing | PTBP1_HUMAN, TRA2B_RAT, ROA1_DROME, SRSF4_MOUSE, HNRPK_CHICK |
| cellular biogenic amine metabolic process | KFA_SALSA, KFA_DANRE, SAT2_BOVIN, SPEE_HUMAN |
| cellular amine metabolic process | KFA_SALSA, CSUP_DROME, KFA_DANRE, SAT2_BOVIN, SPEE_HUMAN |
| regulation of mRNA splicing, via spliceosome | PTBP1_HUMAN, TRA2B_RAT, ROA1_DROME, SRSF4_MOUSE, HNRPK_CHICK, KHDR3_RAT |
| extracellular structure organization | PRDX4_MOUSE, CO1A2_RAT, CO4A1_CAEEL, FLNA_DROME, COBA1_HUMAN, PXDN_DROME, CO3A1_MOUSE, PPN_DROME |
| alpha-amino acid metabolic process | PRDX4_MOUSE, SERA_MOUSE, PDIA1_PONAB, SERB_MOUSE, SPS1_DROME, KFA_SALSA, GCST_HUMAN, KFA_DANRE, PUR4_HUMAN |
| nucleotide biosynthetic process | ANPRB_BOVIN, KFA_SALSA, KFA_DANRE, DUT_HUMAN, PUR9_CHICK, PUR6_DROME, PUR4_HUMAN, TYSY_MOUSE |
| DNA replication | CAF1B_HUMAN, MCM7_HUMAN, RFC5_HUMAN, RFA2_MOUSE, DUT_HUMAN, PSF1_BOVIN, MCM5A_XENLA, MCM2_XENTR, SETMR_HUMAN |
| **FER24 upregulated** | |
| positive regulation of calcineurin-NFAT signaling cascade | SL9A1_HUMAN, CIB1_HUMAN, CIB1_SHEEP |
| regulation of male germ cell proliferation | PRDX4_MOUSE, CIB1_HUMAN, CIB1_SHEEP |
| positive regulation of cholesterol efflux | ABCAC_HUMAN, ABCA1_HUMAN, LRP1_HUMAN |
| cell volume homeostasis | AQP4_BOVIN, S12A2_HUMAN, AQP_DROME, CLCN3_MOUSE, ANXA7_BOVIN |
| water homeostasis | ABCAC_HUMAN, AQP4_BOVIN, AQP_DROME, ANXA7_BOVIN |
| hydrogen peroxide catabolic process | PXDN_HUMAN, PXDN_DROME, PERC_AEDAE, PERC_ANOGA |
| phagocytosis, engulfment | ABCA1_HUMAN, GULP1_HUMAN, CLCN3_MOUSE, GULP1_RAT |
| protein hydroxylation | PDIA1_PONAB, P4HA2_HUMAN, P4HA1_CAEEL, P4HA3_BOVIN |
| serine family amino acid biosynthetic process | GGT1_RAT, SERB_MOUSE, CBS_RAT, AGT2_BOVIN |
| retinoid metabolic process | RDH2_RAT, RDH14_MOUSE, AL1A2_HUMAN, RDHE2_HUMAN, LRP1_HUMAN, LRP2_HUMAN |
| muscle organ morphogenesis | TITIN_MOUSE, TNNT_DROME, TITIN_HUMAN, COBA1_HUMAN, CO3A1_MOUSE |
| cellular hormone metabolic process | RDH2_RAT, CP17A_CHICK, RDH14_MOUSE, AL1A2_HUMAN, RDHE2_HUMAN, ASMT_BOVIN |
| positive regulation of cell-substrate adhesion | PTN_RAT, VWC2_MOUSE, CIB1_HUMAN, NID1_HUMAN, FLNA_MOUSE, CIB1_SHEEP |
| regulation of reproductive process | PRDX4_MOUSE, CIB1_HUMAN, AQP_DROME, CIB1_SHEEP, IF4G3_MOUSE, ZAN_MOUSE, ACH2_CAEEL |
| cellular amino acid catabolic process | 3HIDH_HUMAN, OAT_HUMAN, FTCD_CHICK, CBS_RAT, ASGL1_XENLA, IVD_CAEEL, AGT2_BOVIN, METK1_HUMAN, AASS_BOVIN |
| extracellular structure organization | CR3L2_DANRE, FLNA_DROME, MMP13_RABIT, PXDN_HUMAN, TENX_HUMAN, PPN_DROME, PRDX4_MOUSE, CO1A2_RAT, COBA1_HUMAN, LRP1_HUMAN, NID1_HUMAN, PXDN_DROME, SPTCA_DROME, CO3A1_MOUSE |
| extracellular matrix organization | PRDX4_MOUSE, CR3L2_DANRE, CO1A2_RAT, COBA1_HUMAN, LRP1_HUMAN, NID1_HUMAN, MMP13_RABIT, PXDN_HUMAN, PXDN_DROME, TENX_HUMAN, CO3A1_MOUSE, PPN_DROME |
| reactive oxygen species metabolic process | RGN_RAT, PRDX4_MOUSE, CBS_RAT, PXDN_HUMAN, CLCN3_MOUSE, PXDN_DROME, PERC_AEDAE, AGT2_BOVIN, PERC_ANOGA |
| glycosylation | B3GN5_PIG, MGT4B_DANRE, GCNT1_MOUSE, TMM59_MOUSE, ALG8_HUMAN, BRE4_CAEBR, PMGT1_HUMAN, D19L1_HUMAN, FUCT1_MOUSE, GALT9_CAEEL, FUCTA_DROME, STT3A_BOVIN, GOGA2_RAT, EDEM2_HUMAN, LRP2_HUMAN |
| glycoprotein metabolic process | B3GN5_PIG, MGT4B_DANRE, GCNT1_MOUSE, TMM59_MOUSE, ALG8_HUMAN, BRE4_CAEBR, PMGT1_HUMAN, D19L1_HUMAN, CANT1_HUMAN, G3ST2_MOUSE, GALT9_CAEEL, COBA1_HUMAN, FUCTA_DROME, STT3A_BOVIN, GOGA2_RAT, EDEM2_HUMAN, LRP2_HUMAN |
| **SEN24 upregulated** | |
| regulation of microvillus organization | PLSI_BOVIN, RPGP1_MOUSE, USH1C_HUMAN |
| cellular hormone metabolic process | RDH11_MOUSE, CP17A_CHICK, RDHE2_HUMAN, RISC_MOUSE, RDH14_HUMAN, S5A1_RAT |
| retinoid metabolic process | RDH11_MOUSE, RDHE2_HUMAN, RISC_MOUSE, RDH14_HUMAN |
| iron ion homeostasis | TRFM_RABIT, MFRN2_HUMAN, SFXN1_PIG, NRAM2_RAT, STEA4_HUMAN |
| transition metal ion transport | TRFM_RABIT, MFRN2_HUMAN, P3C2A_MOUSE, SFXN1_PIG, NRAM2_RAT, STEA4_HUMAN |
| hormone metabolic process | RDH11_MOUSE, CP17A_CHICK, RDHE2_HUMAN, RISC_MOUSE, DUOX2_RAT, RDH14_HUMAN, S5A1_RAT |
| cellular response to growth factor stimulus | FSTL3_HUMAN, ATS7_MOUSE, RPGP1_MOUSE, S5A1_RAT, KCP_HUMAN, CD109_MOUSE, PTPRK_HUMAN, PTN_RAT, CD63_RAT, RAB14_PONAB, COR1B_HUMAN, DOK5_HUMAN, CASP3_PIG, NRP1A_DANRE |
| lipid metabolic process | THAS_RAT, SAP_BOVIN, PLD3_XENLA, P3C2A_MOUSE, SCD5_BOVIN, RISC_MOUSE, S14L2_RAT, PTPM1_RAT, PTPM1_DROME, S5A1_RAT, PLBL2_HUMAN, SERC1_MOUSE, RDH11_MOUSE, CP17A_CHICK, RDHE2_HUMAN, GDPD5_HUMAN, PK3CB_MOUSE, OCTC_MOUSE, FABG_THEMA, RDH14_HUMAN, SAP3_MACFA |
| **FER24 vs FER-TD** | |
| extracellular matrix disassembly | TRFM_RABIT, MMP19_HUMAN |
| plasma membrane organization | PLSI_BOVIN, SPTCB_DROME, TMED2_CRIGR |
| regulation of cell adhesion | TRFM_RABIT, VWC2_MOUSE, LEG3_RABIT |
| single-organism membrane organization | PLSI_BOVIN, GOSR1_MOUSE, SPTCB_DROME, TMED2_CRIGR |
| signaling | MYOM1_APLCA, PTBP1_HUMAN, VWC2_MOUSE, DLGP1_DANRE, TSN18_MOUSE, CAR9_ARATH, SPTCB_DROME, ABR_XENLA, LEG3_RABIT, NEC1_HUMAN |

IDs of the gene products are shown within each gene ontology (GO) term. MAT: mated, FER: fertilization, SEN: senescence, 24: control treatment at 24 °C, TD: heat-shock treatment with temperature decrease.
