# Supplementary material for: Oviducal gland transcriptomics of Octopus maya through physiological stages and the negative effects of temperature on fertilization
Source: PeerJ. 2022 Mar 30;10:e12895. doi: 10.7717/peerj.12895 (PMC8976471; doi:10.7717/peerj.12895)
Supplement: Supplemental Information 2 — E-values belong to the BLASTx searches. [file peerj-10-12895-s002.docx]

Supplementary Table S2. Transcripts from the oviducal gland transcriptome of *O. maya* selected for heatmap and cluster analysis.

| **Transcript ID** | **UniProt ID** | **E-value** | **Protein name** |
| --- | --- | --- | --- |
| **Heatmap 1. Top DEGs among physiological stages** | | | |
| TRINITY_DN7086_c0_g1_i1 | ABR_XENLA | 7.52E-18 | Active breakpoint cluster region-related protein |
| TRINITY_DN7173_c0_g1_i1 | ACH2_CAEEL | 1.01E-10 | Acetylcholine receptor subunit beta-type unc-29 |
| TRINITY_DN43413_c0_g1_i1 | ALG8_HUMAN | 0 | Probable dolichyl pyrophosphate Glc1Man9GlcNAc2 alpha-1,3-glucosyltransferase |
| TRINITY_DN12628_c1_g1_i2 | ANXA7_BOVIN | 1.11E-96 | Annexin A7 |
| TRINITY_DN10217_c0_g1_i1 | AQP_DROME | 6.53E-38 | Aquaporin |
| TRINITY_DN14532_c0_g1_i1 | AQP4_BOVIN | 6.10E-40 | Aquaporin-4 |
| TRINITY_DN13830_c0_g1_i5 | B3GN5_PIG | 2.26E-26 | Lactosylceramide 1,3-N-acetyl-beta-D-glucosaminyltransferase |
| TRINITY_DN13397_c0_g2_i3 | BRE4_CAEBR | 1.41E-74 | Beta-1,4-N-acetylgalactosaminyltransferase bre-4 |
| TRINITY_DN9802_c0_g1_i1 | CBR1_PONAB | 5.90E-98 | Carbonyl reductase [NADPH] 1 |
| TRINITY_DN15112_c0_g1_i1 | CIB1_HUMAN | 1.84E-39 | Calcium and integrin-binding protein 1 |
| TRINITY_DN15112_c0_g1_i2 | CIB1_SHEEP | 3.56E-15 | Calcium and integrin-binding protein 1 |
| TRINITY_DN37588_c0_g1_i1 | D19L1_HUMAN | 0 | Probable C-mannosyltransferase DPY19L1 |
| TRINITY_DN9811_c0_g1_i4 | DLGP1_DANRE | 2.65E-18 | Disks large-associated protein 1 |
| TRINITY_DN32774_c0_g1_i1 | DUOX2_RAT | 4.29E-141 | Dual oxidase 2 |
| TRINITY_DN16072_c0_g1_i2 | EDEM2_HUMAN | 0 | ER degradation-enhancing alpha-mannosidase-like protein 2 |
| TRINITY_DN16442_c0_g2_i1 | FUCTA_DROME | 3.67E-134 | Glycoprotein 3-alpha-L-fucosyltransferase A |
| TRINITY_DN11335_c0_g1_i1 | GALT9_CAEEL | 4.23E-121 | Probable N-acetylgalactosaminyltransferase 9 |
| TRINITY_DN3280_c0_g1_i1 | GCNT1_MOUSE | 1.40E-31 | Beta-1,3-galactosyl-O-glycosyl-glycoprotein beta-1,6-N-acetylglucosaminyltransferase |
| TRINITY_DN10779_c0_g1_i1 | GOGA2_RAT | 1.53E-103 | Golgin subfamily A member 2 |
| TRINITY_DN770_c0_g1_i1 | IF4G3_MOUSE | 8.71E-16 | Eukaryotic translation initiation factor 4 gamma 3 |
| TRINITY_DN11014_c0_g2_i1 | KFA_DANRE | 2.48E-12 | Kynurenine formamidase |
| TRINITY_DN11014_c0_g1_i1 | KFA_SALSA | 5.44E-63 | Kynurenine formamidase |
| TRINITY_DN15097_c0_g1_i1 | LEG3_RABIT | 1.49E-06 | Galectin-3 |
| TRINITY_DN16242_c0_g1_i8 | LRP2_HUMAN | 6.50E-17 | Low-density lipoprotein receptor-related protein 2 |
| TRINITY_DN29933_c0_g1_i1 | MGT4B_DANRE | 7.02E-38 | Alpha-1,3-mannosyl-glycoprotein 4-beta-N-acetylglucosaminyltransferase B |
| TRINITY_DN11848_c0_g1_i1 | NEC1_HUMAN | 0 | Neuroendocrine convertase 1 |
| TRINITY_DN16362_c4_g6_i1 | PERC_AEDAE | 3.43E-09 | Chorion peroxidase |
| TRINITY_DN11201_c0_g1_i1 | PMGT1_HUMAN | 1.56E-53 | Protein O-linked-mannose beta-1,2-N-acetylglucosaminyltransferase 1 |
| TRINITY_DN16150_c1_g1_i4 | PRDX4_MOUSE | 1.49E-125 | Peroxiredoxin-4 |
| TRINITY_DN15812_c1_g1_i12 | PTBP1_HUMAN | 0 | Polypyrimidine tract-binding protein 1 |
| TRINITY_DN6386_c0_g1_i1 | RDH11_MOUSE | 2.91E-38 | Retinol dehydrogenase 11 |
| TRINITY_DN8252_c0_g1_i1 | RDH14_HUMAN | 2.09E-33 | Retinol dehydrogenase 14 |
| TRINITY_DN11094_c0_g1_i1 | RDHE2_HUMAN | 1.30E-95 | Epidermal retinol dehydrogenase 2 |
| TRINITY_DN9492_c0_g1_i2 | RISC_MOUSE | 4.15E-124 | Retinoid-inducible serine carboxypeptidase |
| TRINITY_DN22438_c0_g1_i1 | S5A1_RAT | 1.91E-84 | 3-oxo-5-alpha-steroid 4-dehydrogenase 1 |
| TRINITY_DN14910_c0_g1_i2 | SAT2_BOVIN | 7.52E-11 | Diamine acetyltransferase 2 |
| TRINITY_DN22073_c0_g1_i1 | SPEE_HUMAN | 1.82E-108 | Spermidine synthase |
| TRINITY_DN16117_c0_g2_i2 | SPTCB_DROME | 0 | Spectrin beta chain |
| TRINITY_DN10762_c0_g1_i1 | STT3A_BOVIN | 0 | Dolichyl-diphosphooligosaccharide--protein glycosyltransferase subunit STT3A |
| TRINITY_DN7997_c0_g1_i1 | TMM59_MOUSE | 3.77E-41 | Transmembrane protein 59 |
| TRINITY_DN141_c0_g1_i1 | TSN18_MOUSE | 1.44E-16 | Tetraspanin-18 |
| TRINITY_DN4270_c0_g1_i1 | VWC2_MOUSE | 3.14E-09 | Brorin |
| TRINITY_DN16479_c1_g1_i4 | ZAN_MOUSE | 5.36E-13 | Zonadhesin |
| TRINITY_DN7121_c0_g1_i2 | ZAN_PIG | 1.67E-41 | Zonadhesin |
| **Heatmap 2. Top DEGs between Control and Thermal treatment** | | | |
| TRINITY_DN7086_c0_g1_i1 | ABR_XENLA | 7.52E-18 | Active breakpoint cluster region-related protein |
| TRINITY_DN16071_c0_g1_i1 | ACNT2_MOUSE | 4.90E-74 | Acyl-coenzyme A amino acid N-acyltransferase 2 |
| TRINITY_DN16455_c1_g1_i8 | AGRG6_DANRE | 7.16E-48 | Adhesion G-protein coupled receptor G6 |
| TRINITY_DN16455_c1_g1_i11 | AGRG6_DANRE | 2.28E-55 | Adhesion G-protein coupled receptor G6 |
| TRINITY_DN16455_c1_g1_i12 | AGRG6_DANRE | 1.87E-55 | Adhesion G-protein coupled receptor G6 |
| TRINITY_DN14202_c0_g1_i1 | CAR9_ARATH | 5.82E-09 | Protein C2-DOMAIN ABA-RELATED 9 |
| TRINITY_DN44689_c0_g1_i1 | CPROH_CONVC | 1.10E-24 | Neuropeptide prohormone-4 |
| TRINITY_DN9811_c0_g1_i4 | DLGP1_DANRE | 2.65E-18 | Disks large-associated protein 1 |
| TRINITY_DN15725_c0_g1_i3 | DYH3_MOUSE | 0 | Dynein axonemal heavy chain 3 |
| TRINITY_DN12420_c0_g1_i1 | ENO_DORPE | 0 | Enolase |
| TRINITY_DN16472_c13_g1_i2 | FRIS_LYMST | 3.91E-61 | Soma ferritin |
| TRINITY_DN11469_c0_g1_i1 | GIGA6_CRAGI | 4.38E-15 | Gigasin-6 |
| TRINITY_DN16262_c1_g1_i3 | GOSR1_MOUSE | 4.21E-100 | Golgi SNAP receptor complex member 1 |
| TRINITY_DN16491_c1_g1_i1 | GT2D2_BOVIN | 4.39E-17 | General transcription factor II-I repeat domain-containing protein 2 |
| TRINITY_DN15097_c0_g1_i1 | LEG3_RABIT | 1.49E-06 | Galectin-3 |
| TRINITY_DN12984_c0_g1_i1 | MMP19_HUMAN | 1.39E-48 | Matrix metalloproteinase-19 |
| TRINITY_DN32740_c0_g1_i1 | MYOM1_APLCA | 5.09E-26 | Myomodulin neuropeptides 1 |
| TRINITY_DN11848_c0_g1_i1 | NEC1_HUMAN | 0 | Neuroendocrine convertase 1 |
| TRINITY_DN2512_c0_g1_i1 | OGFD2_XENTR | 3.60E-107 | 2-oxoglutarate and iron-dependent oxygenase domain-containing protein 2 |
| TRINITY_DN3838_c0_g1_i1 | PGAM_DICDI | 2.58E-100 | Probable phosphoglycerate mutase |
| TRINITY_DN16097_c0_g1_i5 | PGSC2_DROSI | 4.65E-50 | Peptidoglycan-recognition protein SC2 |
| TRINITY_DN16325_c5_g1_i1 | PLSI_BOVIN | 0 | Plastin-1 |
| TRINITY_DN15812_c1_g1_i12 | PTBP1_HUMAN | 0 | Polypyrimidine tract-binding protein 1 |
| TRINITY_DN15620_c0_g1_i2 | PYR1_SQUAC | 0 | CAD protein [Includes: Glutamine-dependent carbamoyl-phosphate synthase |
| TRINITY_DN11094_c0_g1_i1 | RDHE2_HUMAN | 1.30E-95 | Epidermal retinol dehydrogenase 2 |
| TRINITY_DN15699_c7_g1_i3 | SC6A9_XENLA | 0 | Sodium- and chloride-dependent glycine transporter 1 |
| TRINITY_DN14369_c0_g1_i2 | SCRY3_ENTDO | 9.52E-151 | S-crystallin 3 |
| TRINITY_DN8587_c0_g1_i2 | SL172_CAEEL | 5.93E-22 | Uncharacterized transporter slc-17.2 |
| TRINITY_DN16117_c0_g2_i2 | SPTCB_DROME | 0 | Spectrin beta chain |
| TRINITY_DN16292_c0_g1_i5 | SSPO_CHICK | 0 | SCO-spondin |
| TRINITY_DN38095_c0_g1_i1 | TMED2_CRIGR | 1.04E-103 | Transmembrane emp24 domain-containing protein 2 |
| TRINITY_DN37643_c0_g1_i1 | TRFM_RABIT | 1.36E-63 | Melanotransferrin |
| TRINITY_DN141_c0_g1_i1 | TSN18_MOUSE | 1.44E-16 | Tetraspanin-18 |
| TRINITY_DN15667_c0_g1_i1 | VWA3B_HUMAN | 8.06E-103 | von Willebrand factor A domain-containing protein 3B |
| TRINITY_DN4270_c0_g1_i1 | VWC2_MOUSE | 3.14E-09 | Brorin |
| TRINITY_DN16464_c0_g1_i2 | YL154_YEAST | 9.17E-08 | Uncharacterized protein YLR154C-G |
| **Heatmap 3. Biological regulation genes** | | | |
| TRINITY_DN13742_c0_g1_i1 | 3BHS_PIG | 2.19E-50 | 3 beta-hydroxysteroid dehydrogenase/Delta 5-->4-isomerase |
| TRINITY_DN6203_c0_g1_i1 | 5HT1R_DROME | 1.15E-29 | 5-hydroxytryptamine receptor 1 |
| TRINITY_DN2308_c0_g1_i1 | 5HTR_LYMST | 6.55E-34 | 5-hydroxytryptamine receptor |
| TRINITY_DN37457_c0_g1_i1 | CP1_APLCA | 3.36E-15 | Cerebral peptide 1 (APGW-amide) |
| TRINITY_DN12488_c0_g1_i1 | CP17A_CHICK | 2.75E-99 | Steroid 17-alpha-hydroxylase/17,20 lyase |
| TRINITY_DN27593_c0_g1_i1 | CR3L2_DANRE | 6.62E-38 | Cyclic AMP-responsive element-binding protein 3-like protein 2 |
| TRINITY_DN22347_c0_g1_i1 | CR3L4_XENTR | 4.40E-24 | Cyclic AMP-responsive element-binding protein 3-like protein 4 |
| TRINITY_DN15626_c0_g1_i4 | CREM_CANLF | 6.86E-46 | cAMP-responsive element modulator |
| TRINITY_DN41804_c0_g1_i1 | CRFR1_RAT | 8.85E-18 | Corticotropin-releasing factor receptor 1 |
| TRINITY_DN30186_c0_g1_i1 | CRFR2_RAT | 3.17E-16 | Corticotropin-releasing factor receptor 2 |
| TRINITY_DN15757_c0_g1_i2 | CSUP_DROME | 8.14E-81 | Protein catecholamines up |
| TRINITY_DN21784_c0_g1_i1 | DH12B_DANRE | 5.93E-23 | Very-long-chain 3-oxoacyl-CoA reductase-B |
| TRINITY_DN27073_c0_g1_i1 | DHB2_MOUSE | 3.54E-23 | Estradiol 17-beta-dehydrogenase 2 |
| TRINITY_DN23419_c0_g1_i1 | DHB2_RAT | 4.27E-25 | Estradiol 17-beta-dehydrogenase 2 |
| TRINITY_DN15284_c0_g1_i2 | DHB8_CANLF | 6.85E-85 | Estradiol 17-beta-dehydrogenase 8 |
| TRINITY_DN12903_c1_g1_i1 | DOPR1_DROME | 5.00E-104 | Dopamine receptor 1 |
| TRINITY_DN33758_c0_g1_i1 | DOPR2_DROME | 8.19E-72 | Dopamine receptor 2 |
| TRINITY_DN11849_c0_g1_i1 | FANA_HELAS | 3.36E-140 | FMRFamide-activated amiloride-sensitive sodium channel |
| TRINITY_DN27125_c0_g1_i1 | FMRF_DORPE | 9.43E-63 | FMRFamide-related neuropeptides |
| TRINITY_DN21361_c0_g1_i1 | GNRHR_OCTVU | 2.73E-40 | Gonadotropin-releasing hormone receptor |
| TRINITY_DN14636_c0_g1_i2 | KAPR_BLAEM | 5.29E-16 | cAMP-dependent protein kinase regulatory subunit |
| TRINITY_DN44001_c0_g1_i1 | KAPR2_DROME | 4.03E-32 | cAMP-dependent protein kinase type II regulatory subunit |
| TRINITY_DN29094_c0_g1_i1 | MPRA_CYNNE | 9.17E-17 | Membrane progestin receptor alpha |
| TRINITY_DN32740_c0_g1_i1 | MYOM1_APLCA | 5.09E-26 | Myomodulin neuropeptides 1 |
| TRINITY_DN10537_c0_g1_i1 | PIBF1_HUMAN | 4.32E-37 | Progesterone-induced-blocking factor 1 |
| TRINITY_DN6408_c0_g1_i1 | ST1E1_BOVIN | 2.27E-45 | Estrogen sulfotransferase |
| TRINITY_DN34294_c0_g1_i1 | ST1E1_HUMAN | 5.19E-13 | Estrogen sulfotransferase |

E-values belong to the BlastX searches
