# Supplementary material for: Oviducal gland transcriptomics of Octopus maya through physiological stages and the negative effects of temperature on fertilization
Source: PeerJ. 2022 Mar 30;10:e12895. doi: 10.7717/peerj.12895 (PMC8976471; doi:10.7717/peerj.12895)

Supplementary Figure S1. Scatter plot with linear regression for RNA-seq (x) vs RT-qPCR (y) relative gene expression values (in Log2) in oviducal gland samples of *O. maya*. The Spearman correlation for both variables was significant (P = 0.009).


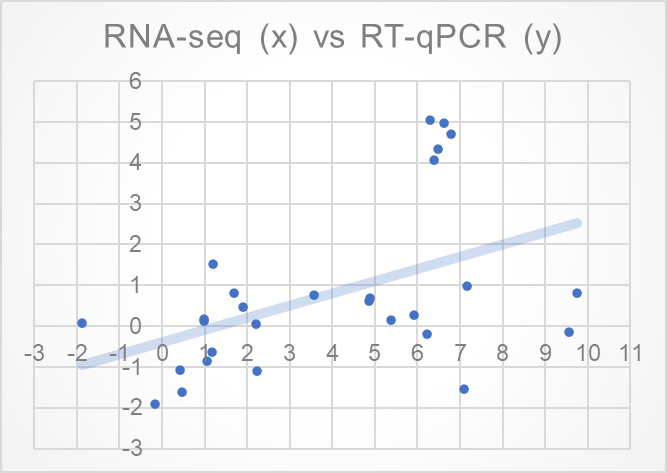

Supplement: Supplemental Information 3 — The Spearman correlation for both variables was significant (P = 0.009). [file peerj-10-12895-s003.docx]
